# Stability of mental motor-imagery classification in EEG depends on the choice of classifier model and experiment design, but not on signal preprocessing

**DOI:** 10.3389/fncom.2023.1142948

**Published:** 2023-04-26

**Authors:** Martin Justinus Rosenfelder, Myra Spiliopoulou, Burkhard Hoppenstedt, Rüdiger Pryss, Patrick Fissler, Mario della Piedra Walter, Iris-Tatjana Kolassa, Andreas Bender

**Affiliations:** ^1^Institute of Psychology and Education, Clinical and Biological Psychology, Ulm University, Ulm, Germany; ^2^Therapiezentrum Burgau, Burgau, Germany; ^3^Knowledge Management and Discovery Lab, Faculty of Computer Science, Otto-von-Guericke-University Magdeburg, Magdeburg, Germany; ^4^Institute of Databases and Information Systems, Ulm University, Ulm, Germany; ^5^Institute of Clinical Epidemiology and Biometry, University of Würzburg, Würzburg, Germany; ^6^Psychiatric Services Thurgau, Münsterlingen, Switzerland; ^7^University Hospital for Psychiatry and Psychotherapy, Paracelsus Medical University, Salzburg, Austria; ^8^Faculty 2: Biology/Chemistry, University of Bremen, Bremen, Germany; ^9^Department of Neurology, University of Munich, Munich, Germany

**Keywords:** motor-imagery, encephalography, machine learning, support vector machine, k-nearest neighbors, classification, classifier, accuracy

## Abstract

**Introduction:**

Modern consciousness research has developed diagnostic tests to improve the diagnostic accuracy of different states of consciousness via electroencephalography (EEG)-based mental motor imagery (MI), which is still challenging and lacks a consensus on how to best analyse MI EEG-data. An optimally designed and analyzed paradigm must detect command-following in all healthy individuals, before it can be applied in patients, e.g., for the diagnosis of disorders of consciousness (DOC).

**Methods:**

We investigated the effects of two important steps in the raw signal preprocessing on predicting participant performance (F1) and machine-learning classifier performance (area-under-curve, AUC) in eight healthy individuals, that are based solely on MI using high-density EEG (HD-EEG): artifact correction (manual correction with vs. without Independent Component Analysis [ICA]), region of interest (ROI; motor area vs. whole brain), and machine-learning algorithm (support-vector machine [SVM] vs. k-nearest neighbor [KNN]).

**Results:**

Results revealed no significant effects of artifact correction and ROI on predicting participant performance (F1) and classifier performance (AUC) scores (all *p*s > 0.05) in the SVM classification model. In the KNN model, ROI had a significant influence on the classifier performance [*F*_(1,8.939)_ = 7.585, *p* = 0.023]. There was no evidence for artifact correction and ROI selection changing the prediction of participants performance and classifier performance in EEG-based mental MI if using SVM-based classification (71–100% correct classifications across different signal preprocessing methods). The variance in the prediction of participant performance was significantly higher when the experiment started with a resting-state compared to a mental MI task block [*X*^2^_(1)_ = 5.849, *p* = 0.016].

**Discussion:**

Overall, we could show that classification is stable across different modes of EEG signal preprocessing when using SVM models. Exploratory analysis gave a hint toward potential effects of the sequence of task execution on the prediction of participant performance, which should be taken into account in future studies.

## 1. Introduction

Mental motor-imagery (MI) is the engagement in mentally performing a motoric task (Milton et al., 2008, e.g., playing tennis or swimming). Such tasks can be used in the field of sports (Schack et al., 2014) or to assess the cognitive performance in severely brain-injured patients (Stender et al., 2014; Engemann et al., 2018), making use of event-related desynchronization (ERD) to reliably detect higher cognitive functioning in brain-injured patients (Cruse et al., 2011, 2012b). Detecting MI task performance reliably in healthy people is mandatory for a diagnostic tool estimating covert awareness in brain-injured patients, who cannot react openly on a task. In a landmark study (Goldfine et al., 2011) on mental MI, the authors were able to prove conscious modulations of electrical brain activity time-locked to an active mental or resting-state condition in all healthy controls. However, these modulations were inconsistent. Therefore, we conclude that there is a concern on whether or not it is possible to reliably distinguish between an active and a resting-state condition in a mental MI paradigm when testing healthy persons, i.e., before considering patients with severe brain injuries. Technically, the detection of a stable mental MI brain state seems to depend highly on the signal processing, classification routine and statistical analyses used, as reported in a re-analysis of mental MI data (Henriques et al., 2016). Therefore, in this work, we re-visit the potential of the MI paradigm in healthy individuals, investigating four distinct research questions (RQ).

We first investigate two very critical issues when analyzing EEG data quantitatively: recognition and rejection of artifacts and the selection of the electrode space. Visual inspection of the EEG signal by trained researchers, together with manual removal of artifact-ridden periods of signal, is a common method to remove contaminated channels (Cruse et al., 2011, 2012a), or trails (Cruse et al., 2012b) from the recording. This method of artifact rejection can be applied to clear-cut artifacts, such as eye blinks or movements, but myogenic activity, which is often mixed with brain activity of interest (McMenamin et al., 2010), cannot be removed from the signal with this strategy. Independent component analysis (ICA) serves as a powerful tool in disentangling myogenic from brain activity. The ICA unmixes the data into independent components, which are then classified as myogenic or genuine brain activity by visual inspection. Misclassification by trained experts might, however, be responsible for the limited performance of an ICA (Olbrich et al., 2011). About one third of EEG classification studies uses manual artifact cleaning, followed by no artifact removal, and automated procedures (e.g., ICA; Craik et al., 2019). Recent work suggested a combination of semi-automatic thresholding and ICA artifact detection and removal in order to clean EEG data from non-cortical activity (Chennu et al., 2017). In EEG-based mental MI, the space of electrodes has been continuously reduced from publications using 25 electrodes spanning the primary motor cortex in the beginning (Cruse et al., 2011, 2012a), down to four electrodes (Cruse et al., 2012b). Further research showed that MI tasks are not restricted to neuronal activity in the motor cortex, and selecting electrodes over the whole cortex might capture additional relevant activity in participants who do not show relevant primary motor cortex activity (Krusienski et al., 2012; Höller et al., 2013b). A methodological study found that depending on the cross-validation strategy, a larger electrode set (63 electrodes) performed better than a small subset (18 electrodes; Henriques et al., 2016). Therefore, under RQ1 we investigate the effect of artifact rejection methods applying an ICA on top of the manual detection and two electrode sets of different size on participant performance.

We then focus on the machine-learning methods used to detect patterns in the extracted EEG signal features. There are recent approaches using deep learning algorithms (Craik et al., 2019; Liu et al., 2021; Zhang et al., 2021; Lomelin-Ibarra et al., 2022). However, in biomedical applications with many features to take into account, still linear classifiers (e.g., SVM) or KNN classifiers are widely used (Mahmoudi and Shamsi, 2018). SVM classifiers are robust, insensitive against the curse of dimensionality, and easier to compute than neural networks (Aggarwal and Chugh, 2019). KNN classifiers are computationally more demanding, but at the same time not that much sensitive to overfitting as SVM classifiers (Aggarwal and Chugh, 2022). In a mental MI study with twenty healthy individuals, the authors found a variation in detection of command-following based on different signal extraction and cross-validation techniques (Henriques et al., 2016). In a two-class classification problem, the authors (Höller et al., 2013b) tested a KNN model against a SVM model and found only small differences in classification accuracy with false-discovery rate (FDR) correction in healthy individuals. This means that both models could be used to inform classification accuracy adequately. However, it is not clear how the treatment of the raw signal before classification influences the performance of a classifier, as referred to in RQ2.

RQ1 How do artifact removal and selection of electrode space (ROI) affect the representation of participant performance in separating between the mental MI and resting-state task?

RQ2 How does the data preprocessing described in RQ1 affect the performance of a classifier that has been trained to separate between the mental MI and resting-state task?

Furthermore, we looked at the number of correctly classified instances per preprocessing method, asking:

RQ3 How does the number of correctly classified instances (mental MI and resting-state tasks) vary across signal preprocessing methods?

We also tested (a) if the order of tasks in the first two blocks (mental MI—resting-state vs resting-state—mental MI) of the experiment could influence the performance of the machine-learning model and (b) if starting with two blocks of the same condition (mental MI—mental MI or resting-state—resting-state), or two blocks of different conditions (mental MI—resting-state or resting-state—mental MI), influences the performance of the machine-learning model.

RQ4a Does the order of tasks in the first two blocks (mental MI—resting-state vs. resting-state—mental MI) of the experiment influence the performance of the machine-learning model?
RQ4b Does starting the experiment with two blocks of the same condition (mental MI—mental MI or resting-state—resting-state) or two blocks of different conditions (mental MI—resting-state or resting-state—mental MI), influences the performance of the machine-learning model?

## 2. Materials and methods

### 2.1. Participants

Participants for this study were recruited for a randomized controlled trial on the recovery from disorders of consciousness conducted at the Therapiezentrum Burgau (Burgau, Germany), serving as healthy control participants. The Therapiezentrum Burgau is a neurological rehabilitation center specialized for DOC patients. Ethics approval has been granted by the Ethics Committee of the Medical Faculty of the University of Munich (Project-Nr. 560-15). All experiments have been conducted respecting the relevant guidelines and regulations as mentioned in the Declaration of Helsinki. In total, 11 participants have been recruited for the study. Data from three participants had to be excluded due to technical problems during data acquisition. In total, data from eight participants was analyzed for this paper (age: *M* = 43.00, *SD* = 15.24; ratio male:female = 2:6). All participants provided oral and written informed consent for participation. From all participants, demographic data was recorded together with the mental MI paradigm. In one participant (P8), two models had to be excluded as data from the manual artifact correction in both electrode spaces were not enough for classification after artifact removal. Data from this case is referred to as “NA” in Tables 1, 2. None of the participants reported psychological or neurological diseases. Participants reported prior to the experiment that they understood the procedure and were asked to follow the instructions as closely as possible, and to stay focused during the MI tasks.

**Table 1:** Overview of support vector machine (SVM) model parameters.

**Participant**	**ROI**	**Cleaning**	**Accuracy**	**95% CI**	***p*-value**	**F1**	**Sensitivity**	**Specificity**	**Precision**	**AUC**	**Brier score**
P1	MA	ICA	0.610	[0.516 0.699]	**0.021**	0.629	0.600	0.623	0.661	0.61	0.253
P2	MA	ICA	0.766	[0.667 0.847]	**< 0.001**	0.789	0.789	0.738	0.789	0.763	0.247
P3	MA	ICA	0.558	[0.461 0.651]	0.259	0.603	0.567	0.544	0.644	0.554	0.255
P4	MA	ICA	0.827	[0.740 0.894]	**<0.001**	0.813	0.796	0.855	0.830	0.827	0.248
P5	MA	ICA	0.670	[0.577 0.753]	**<0.001**	0.688	0.652	0.692	0.729	0.669	0.254
P6	MA	ICA	0.964	[0.910 0.990]	**<0.001**	0.964	0.930	1	1	0.966	0.251
P7	MA	ICA	0.620	[0.522 0.712]	**0.016**	0.624	0.576	0.674	0.680	0.624	0.256
P8	MA	ICA	0.565	[0.466 0.660]	0.211	0.525	0.619	0.530	0.456	0.571	0.269
					Correctly classified						
					instances: 75%						
P1	MA	MAN	0.653	[0.559 0.738]	**0.001**	0.696	0.618	0.714	0.797	0.653	0.271
P2	MA	MAN	0.759	[0.667 0.836]	**<0.001**	0.759	0.774	0.746	0.746	0.76	0.250
P3	MA	MAN	0.617	[0.522 0.706]	**0.012**	0.569	0.674	0.583	0.492	0.621	0.269
P4	MA	MAN	0.868	[0.792 0.924]	**<0.001**	0.865	0.842	0.895	0.889	0.869	0.25
P5	MA	MAN	0.633	[0.541 0.719]	**0.004**	0.633	0.633	0.633	0.663	0.663	0.25
P6	MA	MAN	0.957	[0.901 0.986]	**<0.001**	0.957	0.933	0.982	0.983	0.957	0.251
P7	MA	MAN	0.676	[0.578 0.764]	**<0.001**	0.605	0.703	0.662	0.531	0.667	0.262
P8	MA	MAN	NA	NA	NA	NA	NA	NA	NA	NA	NA
					Correctly classified						
					instances: 100%						
P1	WB	ICA	0.661	[0.563 0.749]	**0.001**	0.648	0.694	0.633	0.607	0.662	0.254
P2	WB	ICA	0.747	[0.648 0.831]	**<0.001**	0.797	0.723	0.800	0.887	0.729	0.263
P3	WB	ICA	0.737	[0.646 0.815]	**<0.001**	0.643	0.931	0.671	0.491	0.729	0.302
P4	WB	ICA	0.838	[0.753 0.903]	**<0.001**	0.821	0.886	0.803	0.765	0.836	0.254
P5	WB	ICA	0.591	[0.490 0.685]	0.078	0.469	0.679	0.558	0.359	0.593	0.307
P6	WB	ICA	0.943	[0.880 0.979]	**<0.001**	0.944	0.911	0.980	0.981	0.943	0.251
P7	WB	ICA	0.561	[0.457 0.661]	0.266	0.557	0.529	0.596	0.587	0.563	0.252
P8	WB	ICA	0.723	[0.625 0.807]	**<0.001**	0.754	0.796	0.638	0.717	0.724	0.245
					Correctly classified						
					instances: 75%						
P1	WB	MAN	0.580	[0.483 0.673]	0.108	0.647	0.558	0.629	0.768	0.580	0.285
P2	WB	MAN	0.717	[0.621 0.800]	**<0.001**	0.712	0.740	0.696	0.685	0.718	0.251
P3	WB	MAN	0.755	[0.663 0.832]	**<0.001**	0.697	0.886	0.693	0.574	0.751	0.28
P4	WB	MAN	0.836	[0.754 0.900]	**<0.001**	0.824	0.894	0.794	0.764	0.836	0.255
P5	WB	MAN	0.589	[0.492 0.681]	0.072	0.500	0.657	0.558	0.404	0.593	0.289
P6	WB	MAN	0.930	[0.866 0.969]	**<0.001**	0.929	0.929	0.931	0.929	0.93	0.25
P7	WB	MAN	0.608	[0.508 0.700]	**0.033**	0.344	0.733	0.587	0.225	0.578	0.349
P8	WB	MAN	NA	NA	NA	NA	NA	NA	NA	NA	NA
					Correctly classified						
					instances: 71%						

ICA, independent component analysis; MA, motor area; MAN, manual artifact detection; ROI, region of interest; WB, whole-brain area; Accuracy, accuracy level of classification and proportional estimate in exact binomial test with 95% confidence interval (CI) and corresponding p-value. F1 denotes F1 scores indicating participant performance in the classification model. “Correctly classified instances” refers to the relative number of configurations allowing for an accurate prediction of participant performance in the binomial test at level 95%. The p-values of these accurate predictions are expressed in bold letters. AUC, area-under-curve.

**Table 2:** Overview of k-nearest neighbor (KNN) model parameters.

**Participant**	**ROI**	**Cleaning**	**Accuracy**	**95% CI**	***p*-value**	**F1**	**Sensitivity**	**Specificity**	**Precision**	**AUC**	**Brier score**
P1	MA	ICA	0.568	[0.473 0.659]	0.167	0.687	0.539	0.786	0.949	0.568	0.394
P2	MA	ICA	0.670	[0.566 0.764]	**0.001**	0.735	0.662	0.690	0.827	0.652	0.266
P3	MA	ICA	0.531	[0.435 0.625]	0.573	0.619	0.538	0.515	0.729	0.522	0.284
P4	MA	ICA	0.548	[0.447 0.646]	0.378	0.662	0.500	0.917	0.979	0.586	0.435
P5	MA	ICA	0.525	[0.421 0.618]	0.645	0.263	0.588	0.515	0.170	0.525	0.377
P6	MA	ICA	0.631	[0.534 0.720]	**0.008**	0.721	0.564	1	1	0.647	0.386
P7	MA	ICA	0.620	[0.522 0.712]	**0.016**	0.506	0.634	0.613	0.420	0.607	0.273
P8	MA	ICA	0.593	[0.494 0.686]	0.067	0.662	0.589	0.600	0.754	0.583	0.271
					Correctly classified						
					instances: 38%						
P1	MA	MAN	0.525	[0.421 0.618]	0.645	0.678	0.513	1	1	0.525	0.475
P2	MA	MAN	0.667	[0.569 0.754]	**<0.001**	0.705	0.642	0.707	0.782	0.665	0.262
P3	MA	MAN	0.444	[0.351 0.539]	0.263	0.467	0.459	0.426	0.475	0.557	0.25
P4	MA	MAN	0.474	[0.379 0.569]	0.640	0.643	0.474	NA	1	0.5	0.526
P5	MA	MAN	0.633	[0.541 0.719]	**0.004**	0.542	0.722	0.595	0.433	0.633	0.29
P6	MA	MAN	0.583	[0.487 0.674]	0.093	0.704	0.5429	1	1	0.586	0.424
P7	MA	MAN	0.562	[0.462 0.659]	0.241	0.425	0.548	0.568	0.347	0.549	0.278
P8	MA	MAN	NA	NA	NA	NA	NA	NA	NA	NA	NA
					Correctly classified						
					instances: 29%						
P1	WB	ICA	0.569	[0.470 0.663]	0.180	0.701	0.545	0.875	0.982	0.557	0.420
P2	WB	ICA	0.632	[0.526 0.728]	**0.013**	0.733	0.615	0.706	0.906	0.596	0.316
P3	WB	ICA	0.465	[0.371 0.561]	0.512	0.371	0.429	0.486	0.327	0.540	0.263
P4	WB	ICA	0.486	[0.387 0.585]	0.845	0.654	0.486	NA	1	0.5	0.514
P5	WB	ICA	0.533	[0.433 0.631]	0.558	0.380	0.577	0.519	0.283	0.536	0.316
P6	WB	ICA	0.571	[0.471 0.668]	0.172	0.698	0.536	1	1	0.576	0.434
P7	WB	ICA	0.531	[0.427 0.632]	0.614	0.589	0.500	0.594	0.717	0.541	0.291
P8	WB	ICA	0.535	[0.433 0.635]	0.551	0.680	0.575	0.286	0.833	0.535	0.313
					Correctly classified						
					instances: 13%						
P1	WB	MAN	0.518	[0.421 0.613]	0.777	0.667	0.509	0.667	0.964	0.518	0.449
P2	WB	MAN	0.557	[0.457 0.653]	0.285	0.689	0.536	0.778	0.963	0.549	0.414
P3	WB	MAN	0.509	[0.412 0.606]	0.924	0.413	0.500	0.514	0.352	0.506	0.271
P4	WB	MAN	0.509	[0.412 0.606]	0.924	0.671	0.505	1	1	0.509	0.491
P5	WB	MAN	0.509	[0.413 0.605]	0.925	0.546	0.516	0.500	0.579	0.508	0.254
P6	WB	MAN	0.535	[0.439 0.629]	0.512	0.675	0.514	0.857	0.982	0.543	0.45
P7	WB	MAN	0.589	[0.490 0.683]	0.081	0.560	0.549	0.625	0.571	0.587	0.249
P8	WB	MAN	NA	NA	NA	NA	NA	NA	NA	NA	NA
					Correctly classified						
					instances: 0%						

ICA, independent component analysis; MA, motor area; MAN, manual artifact detection; ROI, region of interest; WB, whole-brain area; Accuracy, accuracy level of classification and proportional estimate in exact binomial test with 95% confidence interval (CI) and corresponding p-value. F1 denotes F1 scores indicating participant performance in the classification model. “Correctly classified instances” refers to the relative number of configurations allowing for an accurate prediction of participant performance in the binomial test at level 95%. The p-values of these accurate predictions are expressed in bold letters. AUC, area-under-curve.

### 2.2. Materials from the experiment on mental MI

**Figure 1:**
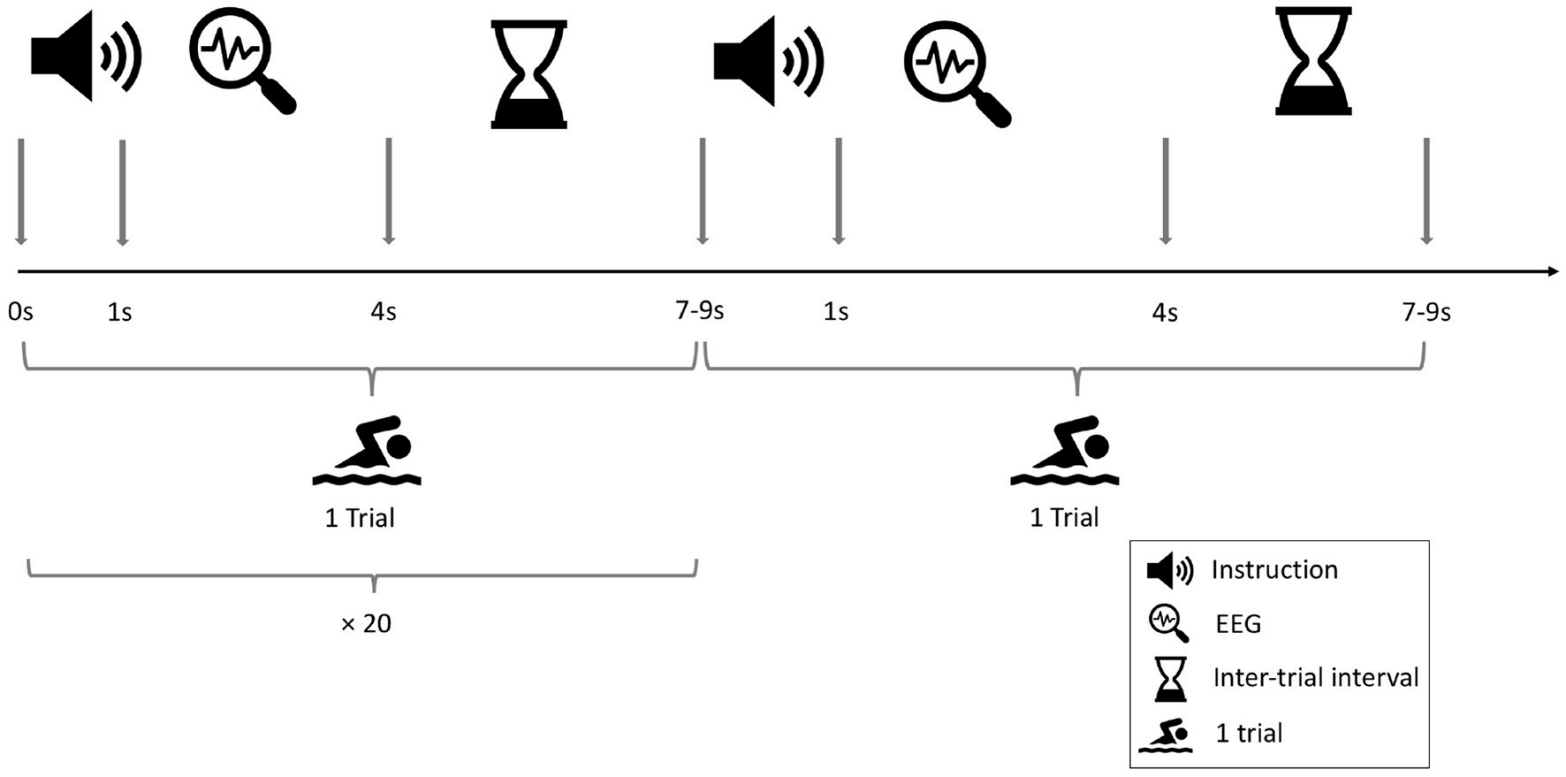
Design of the EEG-based mental motor-imagery experiment. Two consecutive tasks are shown. One block consisted of 20 tasks of the same condition with a single instruction given by the experimenter at the beginning of the block. The procedure consisted of three blocks per condition, which were randomly presented across participants.

The MI task (mentally performing swimming exercises with both arms) was adopted from the work of Goldfine et al. (2011). This task was selected, since it was the only task producing distinct patterns of frequency changes among all healthy controls (desynchronization in mu band following the instruction). Originally, participants had a time window of 13 s to perform the task per trial. However, as in the study (Goldfine et al., 2011), DOC patients had difficulties producing clear patterns of power reduction in the MI tasks, the structure of the task was modified for this study. The resting-state task included the instruction to rest mentally and do nothing. In the original work of Goldfine et al. (2011), the task was openly formulated (“start/stop imagine yourself swimming”). This task leaves the naıve participant with few details on how to perform the task. This in return could potentially negatively affect classification. Thus, the task was modified an adapted to the clear commands as reported in the work of Cruse et al. (2011). The length of each trial was shortened, so that participants had to perform the task for 4 s before the next trigger appeared. The inter-stimulus interval (ISI) between two trials was chosen randomly between 7 and 9 s. The instruction which task to perform (mental MI or resting-state condition) was given once for 20 consecutive trials. These 20 trials always formed a block so that unnecessary task-switching was prevented. Each task block lasted about 3 min. The entire experiment consisted of three blocks per task condition (mental MI and resting-state). The blocks were placed in randomized order. The sequence of task blocks per participant is listed in Supplementary Table 2. Complete randomization of block order ensured that participants could not anticipate which block would come next, which may otherwise have artificially increased performance rates. Between blocks, 1 min breaks allowed the participants to relax and focus again for the upcoming block of trials. The block design was adapted from Cruse et al. (2011). The complete design can be seen in Figure 1.

Auditory triggers announcing a new trial were 1,000 Hz tones with 100 ms duration. Triggers were kept equal among conditions. The verbal instruction for the tasks was given prior to every block by the experimenter (MJR) conducting all HD-EEG sessions. In the mental MI condition participants were asked to imagine one stroke of swimming with both arms as soon as they heard the auditory trigger signal. They were also told to imagine how it would feel performing the task, forcing their mental engagement in the task. In the resting-state condition, participants were instructed to now only listen to the auditory trigger signals. Giving an active verbal instruction in both conditions ensured that participants were equally engaged in both conditions. Even more important, it ensured that the cognitive activation by auditory triggers was equal among conditions.

### 2.3. HD-EEG acquisition and preprocessing

HD-EEG data acquisition was done using an EGI NA400 amplifier with 256 channel input (Electrical Geodesics Inc./Philips Neuro, Eugene, Oregon, USA). EEG data was recorded at 500 Hz sampling rate. The amplifier was connected to a HydroCel GSN sensor net with 256 channels arranged in an extended 10/20 montage. This sensor net works with saline-soaked sponges, mounted into a flexible cap. Impedances were kept below 50 kOhm. Electrodes were online-referenced to the vertex. Original data were converted to raw format prior to signal preprocessing. Preprocessing was done using custom scripts (MATLAB, 2017) and functions from the Fieldtrip toolbox (Oostenveld et al., 2011). Before starting the artifact removal procedure, two different sets of electrodes were created. The first set contained 32 electrodes over the motor cortex ROI, similar to Cruse et al. (2011). The second set of electrodes covered the entire cortex and consisted of 172 electrodes. Offline analysis of data started with applying a band-pass filter between 1–40 and a 50 Hz notch filter removing line noise via applying a discrete Fourier transform. Data were then cut into time-locked segments beginning 1.5 s before the auditory trigger until 4.5 s after the trigger. These segments were referenced against a baseline time-window of −0.5 to 0.0 s, relatively to the trigger signal. For the subsequent artifact correction, it was decided to break the block structure and continue the analyses using a trial-based structure. This means that all trials of the same task (mental MI and resting-state, respectively) were grouped together, keeping their original labels for the machine-learning classification. This was done for two reasons: First, breaking the block structure masked the order of trials to the person involved in data cleaning, thus making the process less subjective. The block membership of trials was disclosed at the end of the data cleaning steps, just before the automated classification. Second, analyzing data in a trial-based structure is recommended by Henriques et al. (2016) for cross-validation processes.

### 2.4. Artifact correction

**Figure 2:**
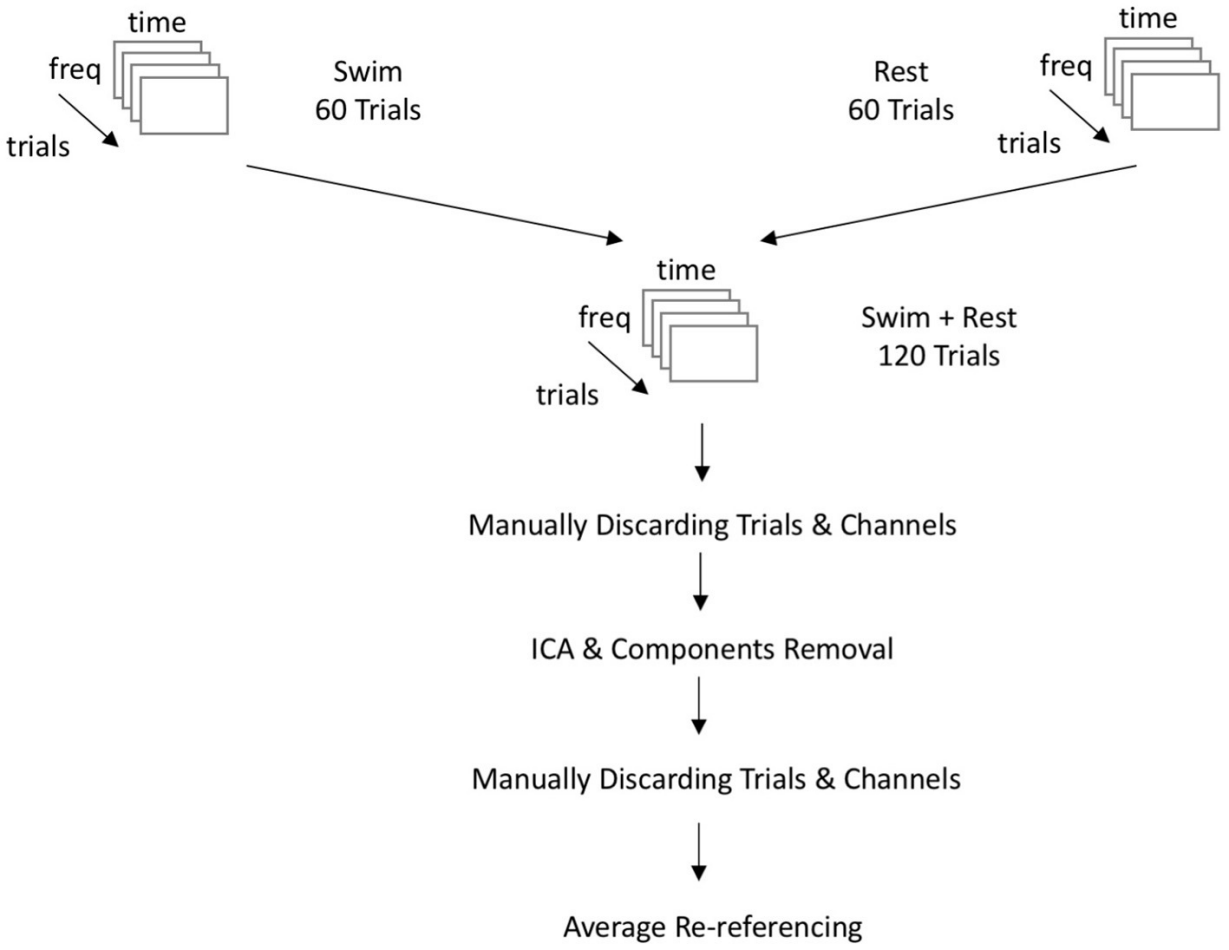
Flowchart of the EEG-data preprocessing. Data is coming from three blocks of both experimental conditions each. All blocks of one condition are appended following their sequence of presentation. freq, frequency domain; time, time domain; Swim, mental MI condition; Rest, resting-state condition; ICA, independent component analysis.

Handling of artifacts in the recordings was two-fold: The first version of artifact correction was done as in the original work by Cruse et al. (2011) and Cruse et al. (2012a,b) on mental MI. The data was cleaned using visual inspection by a trained expert and consecutive removal of noisy trials and channels. Trials and channels were displayed in a matrix based on their variance. Channels and trials with high variance resembling outliers (due to ocular, muscle, or ECG artifacts) were detected visually and consequently removed from the data set. In the second version, data was cleaned from artifacts using a three-step approach adapted from Chennu et al. (2017): first, data was inspected manually and noisy channels and trials were removed as in the first version; second, an Infomax ICA algorithm was run on the data, making it possible to better remove ECG, ocular and muscle artifacts from the data. The Infomax ICA algorithm decomposits the data by maximizing the information which is present in the input data when decomposing the data into independent components (Bell and Sejnowski, 1995). The algorithm is trained on the input data such that the weight matrix is orthogonal to the previous learning step (Makeig et al., 1996). This way, the entropy of the data transformed into ICA matrix space is maximized (Jung et al., 2000). Depending on the ROI, the ICA returned a maximum of 32 (motor cortex) or 172 (whole-brain) components, respectively. Finally a second visual inspection and manual removal of artifact-ridden segments and channels was done in order to exclude remaining noisy epochs or channels. Both versions of artifact correction were done on each data set. The cleaning of the data sets was done by an experienced rater (MJR), who also strongly supervised the second rater (MdPW). In the version with motor area ROI, a mean of 29.40 (*SD* = 3.27) out of 32 electrodes was used for time-frequency analyses. In the version with all cortex electrodes, a mean of 160.53 out of 172 (*SD* = 6.08) channels was used. As for trials, a mean of 111.27 (*SD* = 6.93) trials could be integrated into analyses for the motor area configuration, as well as a mean of 106.87 (*SD* = 5.63) trails in the configuration spanning all cortical electrodes. The number of included good trials in the motor area ROI and the whole brain ROI did not differ significantly [*t*_(26.87)_ = 1.91, *p* = 0.067]. However, the mean number of included trials in the models with ICA-based artifact removal (*n* = 106.6) was significantly different from the number of included trials in the manual artifact removal models [*n* = 111.9, *t*_(25.27)_ = −2.4004, *p* = 0.024]. In the end, the number of included trials per model did not correlate significantly with the participant performance values (F1 scores) in the SVM models (S = 4,663, *p* = 0.844) or the KNN models (S = 5613.1, *p* = 0.185). Supplementary Table 1 gives an overview on the channels and trials used for each analysis model. At the end of the artifact correction procedure, channels were re-referenced to the average of all channels and with channel 257 as implicit reference. The complete approach of data preprocessing and artifact correction is displayed in Figure 2.

### 2.5. Single-subject EEG multivariate analysis

The multivariate analyses were done using (MATLAB, 2017) custom scripts. We tested combinations of two different artifact removal techniques with two ROIs on the performance of a SVM and a KNN model per participant. This procedure resulted in the following signal preprocessing strategies: motor area ROI with manual artifact removal, motor area ROI with manual/ICA artifact removal, whole brain area ROI with manual artifact removal, and whole brain area ROI with manual/ICA-based artifact removal (Supplementary Table 3). After artifact removal and re-referencing, all remaining trials for each task (mental MI and resting-state) were grouped together using the original labels attached to each trial (2 × max. 60 trials). Time-frequency analysis was performed in a multivariate approach. For the time-frequency analysis, a time window of 1–4 s post-stimulus was selected for every trial. To stay close to the original work by Cruse et al. (2011), a frequency range between 7 and 30 Hz of the EEG signal was selected for the time-frequency analysis. Almost the same frequency range had been used before in the analysis of mental motor imagery data (Pfurtscheller et al., 2008), and was kept in a critical re-analysis of the Cruse et al. data (Goldfine et al., 2013). A time-frequency analysis was performed to analyze the signal power at a certain timepoint and frequency, using the *ft_freqanalysis* function from the Fieldtrip toolbox (Oostenveld et al., 2011). Time-frequency representations were reported with power spectra as output and using a Hanning window sliding the data in 0.25 s steps on the entire segment from 1 to 4 s post-stimulus, with each time window lasting 0.5 s. Multitapers were used to control for spectral leakage and resulted in frequency smoothing. This prevents data segments, which are not sampled by the sliding time window from influencing sampled segments of data. Padding the data helped increase the spectral resolution.

### 2.6. EEG-derived features for classification

**Figure 3:**
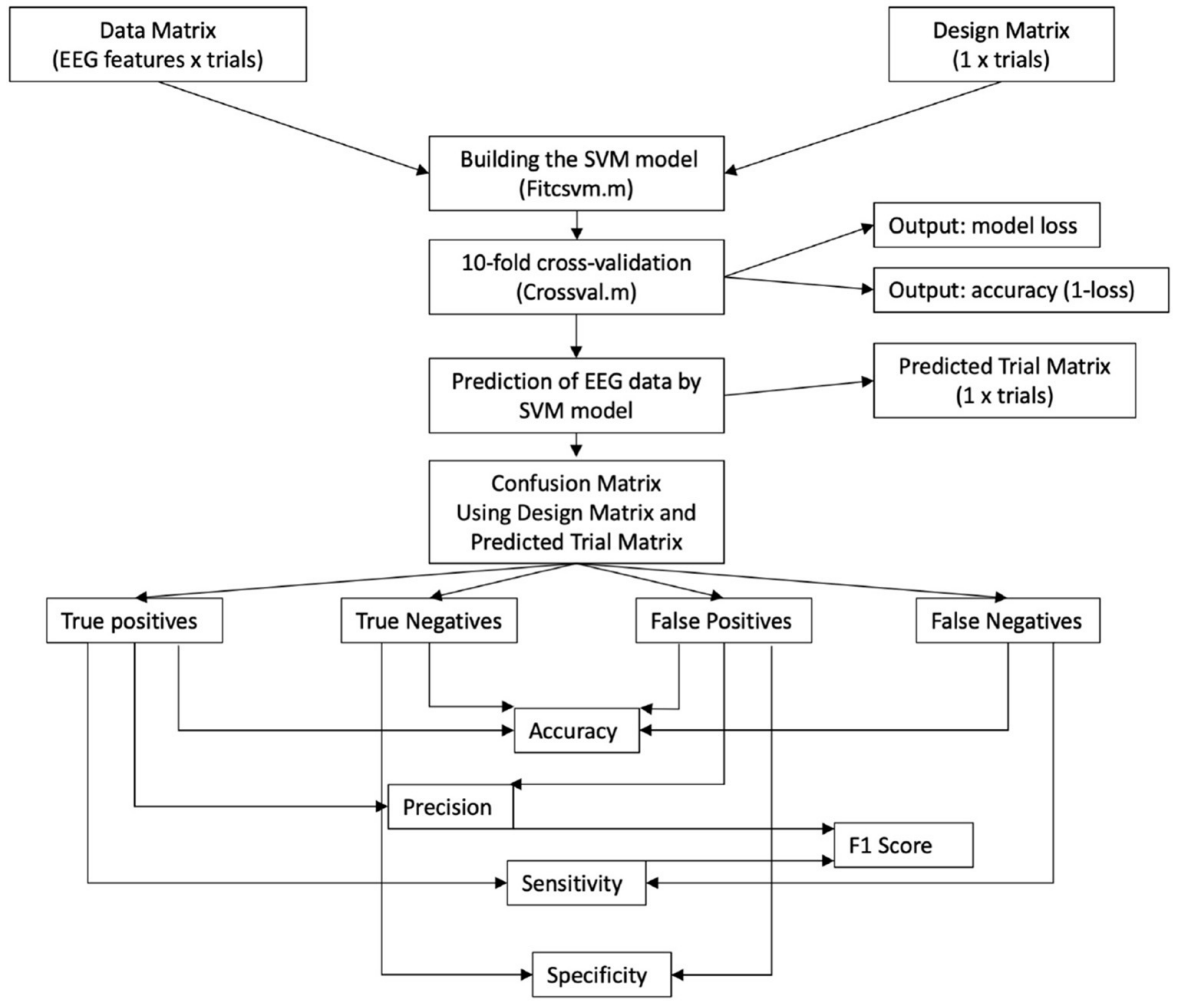
Flowchart of the machine learning approach on preprocessed EEG data—as *trial label* we define the condition each of the single tasks belongs to; it is noted that the number of trial labels varies per participant, because some trials had to be rejected. SVM, support vector machine; F1, indicator for predicting participant performance through a classifier.

Two matrices were fed into the machine-learning pipeline, (see also Figure 3): One matrix (data matrix) contained the EEG features in a time-series per participant from the multivariate EEG analyses. The time-series was organized three-dimensionally (time-points * frequency-bins * channel) and was as large as the number of artifact-free trials for the participant (up to 120 trials). The order of trials in the time-series was stored in a one-dimensional matrix with *n* elements. The other matrix (design matrix) contained the design vector elements, which were defined by two indices for the resting-state and the MI condition, respectively (“1” for mental MI and “2” for resting-state condition). The data and the design matrix were inverted and defined as a vector.

### 2.7. Single-subject classification with machine learning algorithms

For the classification, supervised learning with SVM was chosen. The target variable was the separation between mental MI and resting-state. A trial-based approach was chosen as done by Henriques et al. (2016), breaking the block-structure of the experiment and using all tasks in a row. Random sampling over all trial labels of both tasks was done to create folds for cross-validation. Cross-validation allows for a reliable classification if few trials are available or to make training and testing more effective (Nguyen and Zeigermann, 2018). For *k*-fold cross-validation, the data are split into equal and non-overlapping folds (e.g., *k* = 10 elements), and the validation score is calculated *kn* times. Out of all trials per task group (resting-state and mental MI), the size of the training and test set was determined by dividing the number of entries through the folds, e.g., with 60 entries and 10-folds, the split resulted in the first six entries as test elements and the other 54 as training elements. Training and test elements of both tasks (mental MI and resting-state) were then combined to form a training and test group design matrix for model prediction. The classifier model was built from the training sets of the design matrix and the EEG data matrix (see Figure 3). We performed *k*-fold cross-validation. In each of the *k* runs, we varied the hyperparameters; the performance value of each run is then the value achieved by the best set of hyperparameter values. Once the *k* runs were finished, we computed as performance the average of the performance values over the *k* runs. For each *kn* we computed the performance in each of the folds and averaged over all folds. The *kn* value for which the average over all folds was best is reported as “*kn* with best average over all folds.” Of *k* = 5/10/20 tested for the SVM classifier, *k* = 10 was selected as final parameter, and of *k* = 1/3/5/10/15 options, *k* = 10 was selected for the KNN classifier (see Supplementary Tables 4, 5). Building the model, training, and testing were done on the same data subsets that had been generated for the SVM model. We did not validate this *kn* value on holdout data and did not perform hyperparameter optimization. We did not build any final model over all data.

The prediction with the SVM and the KNN model respectively returned a one-dimensional vector with a label assigned to each trial. Based on the original and the predicted trial labels (for the mental MI and the resting-state task) a confusion matrix was determined. This confusion matrix was built using the design matrix and the predicted-trial matrix, from which true positives, true negatives, false positives, and false negatives were computed with formulae (S1–S5) in custom (MATLAB, 2017) scripts (Supplementary Data 1), based on the function “perfcurve.” Sensitivity and specificity for the classifiers were based on properties of the confusion matrix after classification. Both were computed for each of the four processing methods per participant. Sensitivity and specificity values were computed for every classification separately. All values for the SVM and KNN models can be found in the respective tables (Table 1 for the SVM and Table 2 for the KNN model.).

The mean and standard deviation of F1 respectively AUC scores among the cross-validation iterations define the quality of the classification process. For single-subject classification, scripts were written in MATLAB (version R2017a, 9.2.0.556344).

### 2.8. Predicting classifier performance

Besides the evaluation of the classifier performance within a particular task, we aimed to test the prediction of the classifier regarding the two different tasks presented (mental MI and resting-state). The prediction of classier performance was measured as the fit of original trial labels to the predicted labels, e.g., whether the predicted classes match the original classes of the experiment, based on the task priors presented to the participant and his or her actual brain patterns in response to the tasks. The ratio of fit between real and estimated condition labels per participant was investigated using an exact binomial test in R, implemented in the R package “stats,” with the function “binom.test” (R Core Team, 2019). A *p* < 0.05 tells that the frequency distribution of a dichotomous variable is not random. A statistically significant results in the binomial test was interpreted as a success in the prediction by the classifier and is subsequently defined as *positive* prediction. *P*-values in bold in Tables 1, 2 indicate a significant prediction result as a *positive* result. A non-significant prediction is expressed as a *p* > 0.05 and deemed *negative*.

### 2.9. Rating the success of task prediction among different preprocessing methods and classifiers

The comparison of preprocessing methods and classifiers regarding an accurate task prediction was computed as suggested by Henriques et al. (2016): for each preprocessing strategy (four preprocessing methods, except 2 for P8, for eight participants) we divided the number of models with correctly classified instances (95% level) through the number of total classifications (see Tables 1, 2).

### 2.10. Determining the influence of artifact removal and electrode space on predicting participant performance and classifier performance

The influence of the method of artifact removal and the selection of electrode space on the prediction of participant performance (F1 score) was investigated using a robust ANOVA from the R package “WSR2” (R Core Team, 2019). Since data was not normally distributed as reviewed visually and confirmed by Shapiro-Wilk tests, a non-parametric test (*robust* ANOVA) was applied. The effect of artifact removal and ROI on classifier performance (AUC scores) was investigated using the same statistical model as for participant performance (F1 scores).

### 2.11. Task block configurations

We considered the different configurations of tasks that the participants received in randomized order in an exploratory analysis. Task blocks differed in whether the experiment started with a mental MI vs resting-state task block, and in the adjacency between these blocks: two blocks of the same task or different tasks at the beginning of the experiment (see Supplementary Table 2).

### 2.12. Performance evaluation metrics

Classifier performance was expressed as accuracy and F1-score (or Precision-Recall; Nguyen and Zeigermann, 2018). Accuracy is defined as all correctly classified divided through all instances. The F1 score rates the accuracy of a test in a binary decision task and is expressed as the harmonic ratio between precision and recall. Precision can be defined as the number of correctly identified objects belonging to a class among all identified objects. Recall is the number of correctly identified objects among all objects in the sample (Nguyen and Zeigermann, 2018). It is also referred to as sensitivity. The evaluation of the classifier performance was accomplished with receiver-operating characteristic (ROC) curve. The original labels of all trials of a data set (stored in the design matrix) and the SVM/KNN-model predicted labels (stored in the predicted trial matrix) were re-structured as column vectors. These two vectors were passed to the “roc” function in RStudio Team (2020) (version 2022.02.3), and the area-under-curve (AUC) was derived from the fitted ROC curve. AUC values were determined for every single data set (per participant) using a custom R script and the R package “AUC” (Ballings and Van den Poel, 2013). It should be noticed that classifier performance was calculated per participant, and is thus relative to each participant. Brier scores were used as calibration measure for the classifier. Brier scores near 0 define good model predictions, whereas values near 1 define an ill-calibrated model. The brier scores were calculated using the “scoring” package in RStudio Team (2020) (version 2022.02.3).

## 3. Results

It is important to note that each classifier was trained on a participant's individual set of MI and resting-state trials, which could vary in number of artifact-free tasks after signal processing. Effects on the prediction of participant performance and classifier performance were tested on a group-level. “Participant” was included as blocking variable in the non-parametric *robust* ANOVA tests.

### 3.1. Effects of artifact removal technique and ROI on predicting participant performance (RQ1)

**Figure 4:**
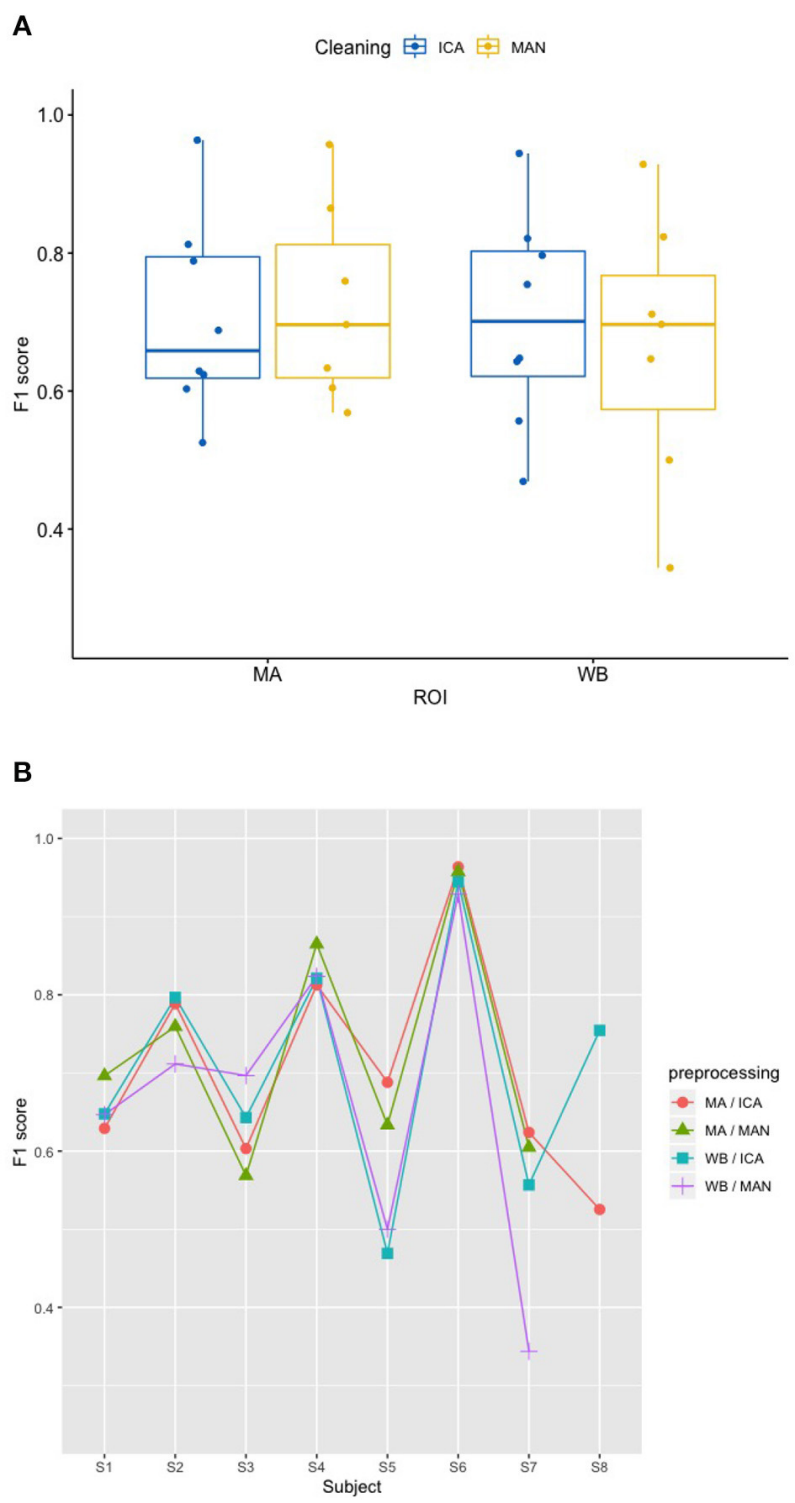
Effect of preprocessing on predicting participant performance in the SVM model. **(A)** No effect of preprocessing (artifact correction method and region of interest) on F1 scores using a support vector machine (SVM) model. The blocking variable “Participant” is ignored in this figure to better show the effect of the preprocessing. Data cleaning was done either by combined manual and ICA artifact correction (ICA) or manually (MAN) on either motor area (MA) or whole-brain (WB) dataset. ROI, region of interest; F1, indicator of participant performance; SVM, support vector machine; ICA, independent component analysis; MAN, manual artifact correction. **(B)** F1 scores across methods and participants in the SVM model show a substantial amount of inter-individual variance. In this figure, the blocking variable “Participant” is accounted for. Methods indicated as follows: MA, motor area ROI; ICA, independent component analysis; WB, whole-brain ROI; MAN, manual artifact correction.

**Figure 5:**
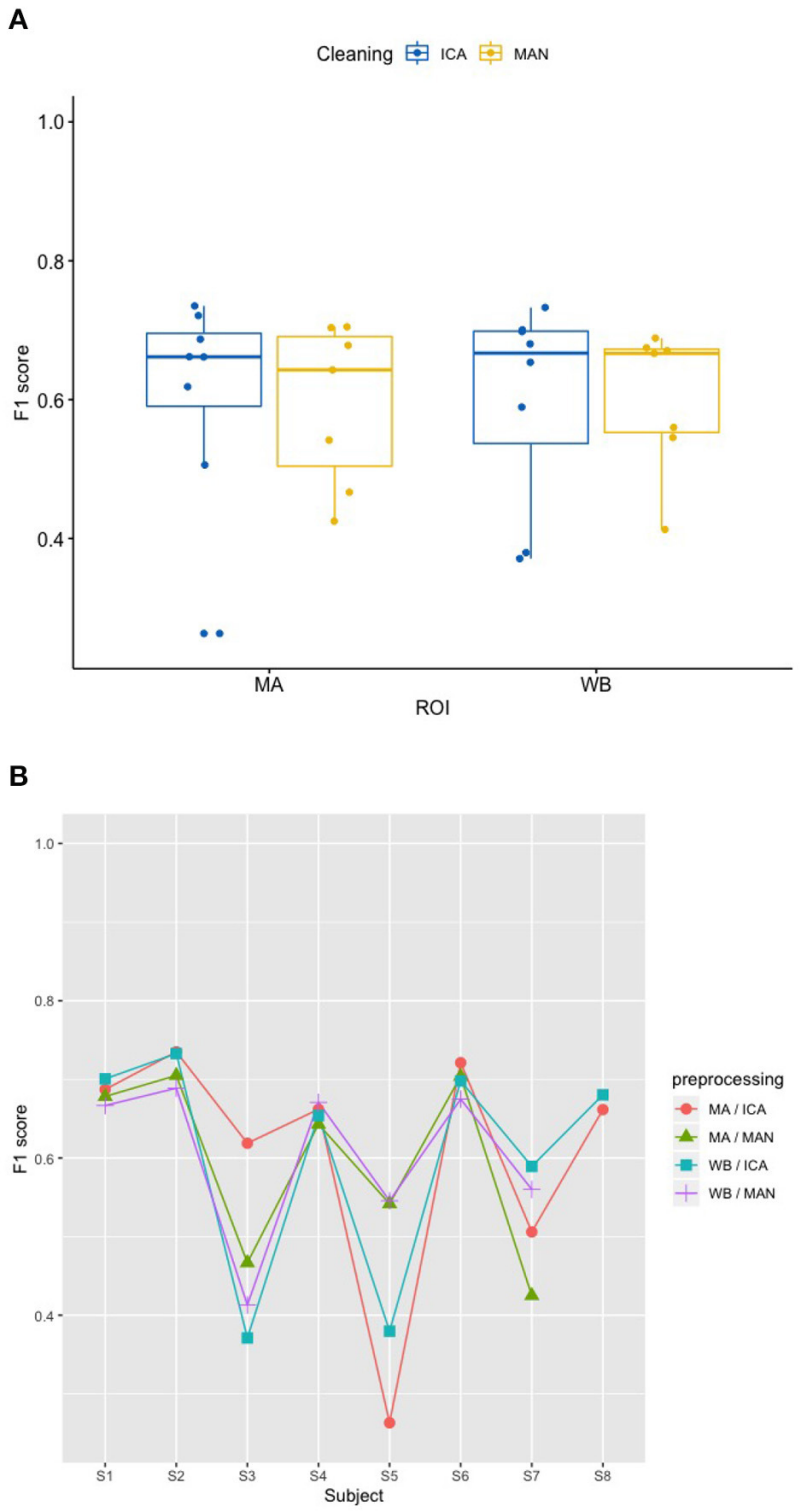
Effect of preprocessing on predicting participant performance in the KNN model. **(A)** No effect of preprocessing (artifact correction method and region of interest) on F1 scores of the classifier using a k-nearest-neighbor (KNN) model. The blocking variable “Participant” is ignored in this figure to better show the effect of the preprocessing. Data cleaning was done either by combined manual and ICA artifact correction (ICA) or manually (MAN) on either motor area (MA) or whole-brain (WB) dataset. ROI, region of interest; F1, indicator of participant performance; KNN, k-nearest-neighbors; ICA, independent component analysis; MAN, manual artifact correction. **(B)** F1 scores across methods and participants in the SVM model show a substantial amount of inter-individual variance. In this figure, the blocking variable “Participant” is accounted for. Methods indicated as follows: MA, motor area ROI; ICA, independent component analysis; WB, whole-brain ROI; MAN, manual artifact correction.

There was no significant effect of neither the method of artifact removal on the quality of the model (F1) [*F*_(1,8.997)_ = 0.002, *p* = 0.967], nor the ROI on the quality of the model (F1) [*F*_(1,9.998)_ = 0.184, *p* = 0.678], see Figure 4A when applying the SVM model. With the KNN model, there was no significant effect of method of artifact removal [*F*_(1,9.928)_ = 0.046, *p* = 0.836] or ROI on F1 scores [*F*_(1,7.89)_ = 0.026, *p* = 0.877, see Figure 5A]. Descriptively, F1 scores derived from the SVM model (Figure 4B) ranged higher than the scores derived from the KNN model (Figure 5B). All values can be found in Table 1 for the SVM model, and in Table 2 for the KNN model as well as in Figure 4B (SVM model) and Figure 5B (KNN model).

### 3.2. Effects of artifact removal technique and ROI on classifier performance (RQ2)

In order to investigate how the classifiers perform among the different configurations of the experiment, Receiver-Operating-Characteristic (ROC) curve analyses were performed on the per-trial pairs of true and SVM-predicted labels. Thus, for each data set, a ROC-curve was fitted on the data and the area under curve (AUC) was computed. As the visual inspection and Shapiro-Wilk tests revealed non-normally distributed data, the *robust* ANOVA was applied for non-parametric testing.

**Figure 6:**
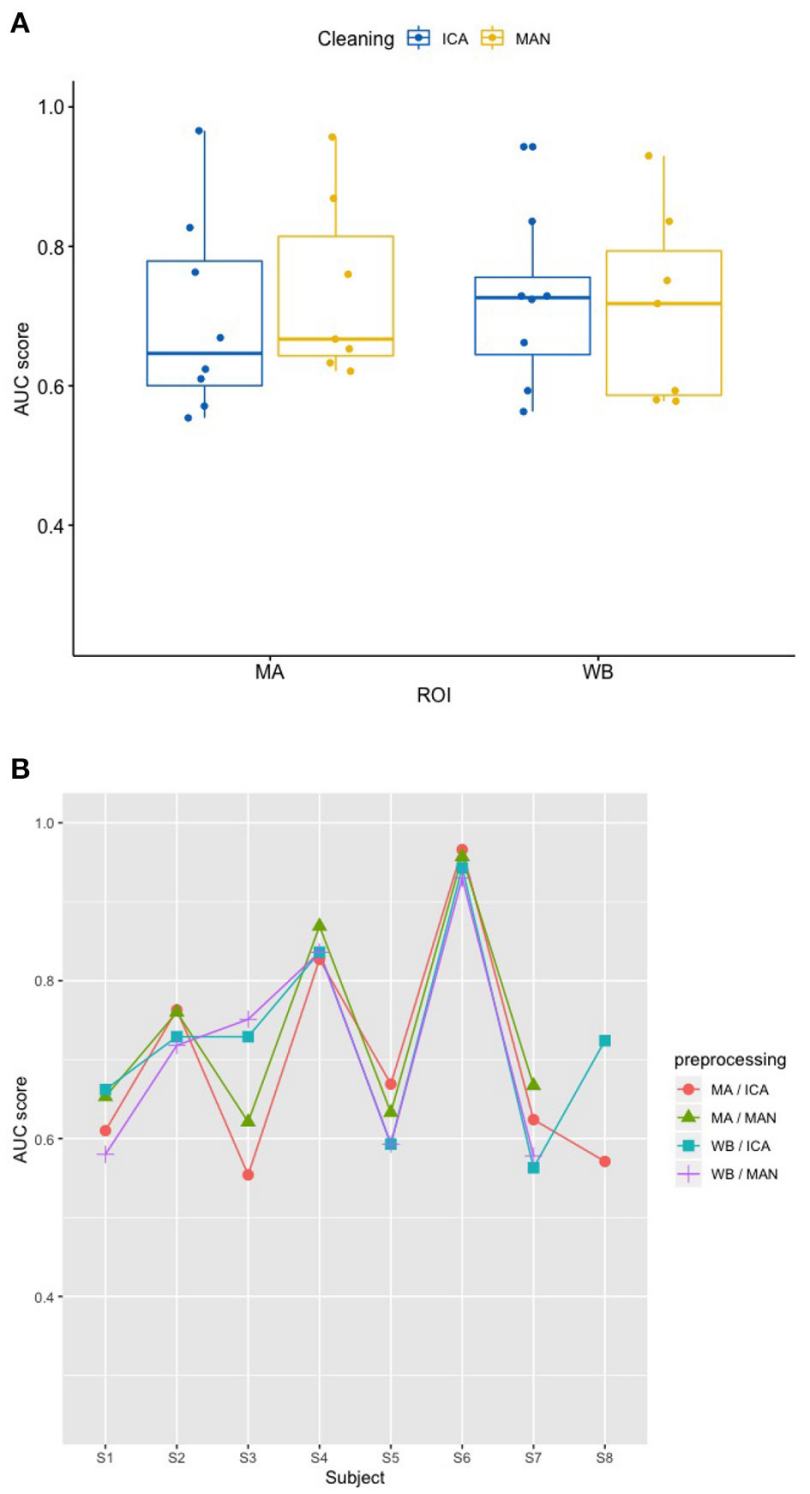
Effect of preprocessing on classifier performance in the SVM model. **(A)** No effect of preprocessing (artifact correction method and region of interest) on AUC scores in the support vector machine (SVM) model. The blocking variable “Participant” is ignored in this figure to better show the effect of the preprocessing. Data cleaning was done either by combined manual and ICA artifact correction (ICA) or manually (MAN) on either motor area (MA) or whole-brain (WB) dataset. ROI, region of interest; F1, indicator of participant performance; SVM, support vector machine; ICA, independent component analysis; MAN, manual artifact correction. **(B)** AUC scores across participants in the SVM model show a substantial amount of inter-individual variance. In this figure, the blocking variable “Participant” is accounted for. Methods indicated as follows: MA, motor area ROI; ICA, independent component analysis; WB, whole-brain ROI; MAN, manual artifact correction.

**Figure 7:**
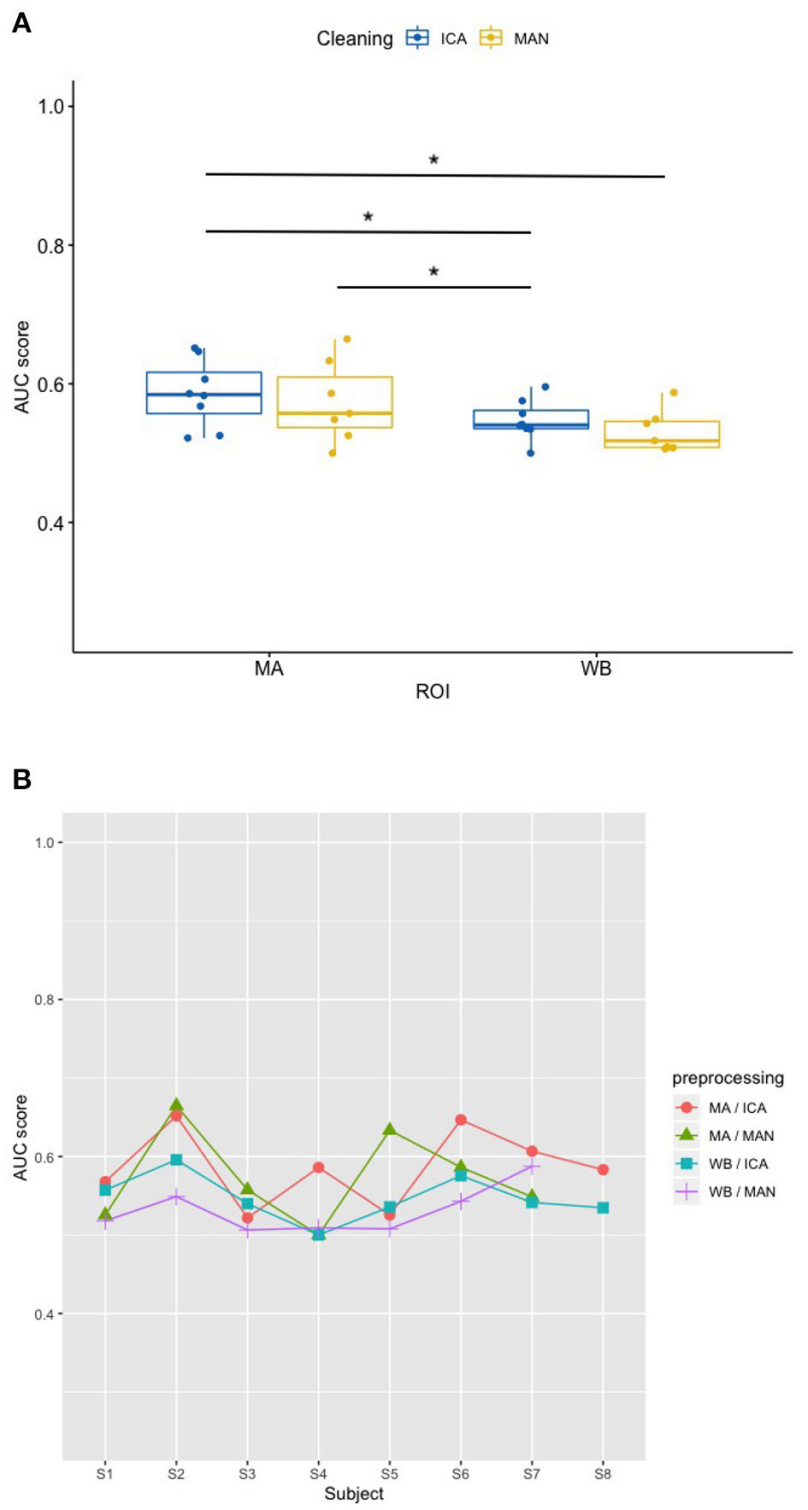
Effect of preprocessing on classifier performance in the KNN model. **(A)** Significant effect of preprocessing (artifact correction method and region of interest) on AUC scores in the k-nearest-neighbor (KNN) model. The blocking variable “Participant” is ignored in this figure to better show the effect of the preprocessing. Data cleaning was done either by combined manual and ICA artifact correction (ICA) or manually (MAN) on either motor area (MA) or whole-brain (WB) dataset. ROI, region of interest; F1, indicator of participant performance; KNN, k-nearest-neighbors; ICA, independent component analysis; MAN, manual artifact correction. ^*^p < 0.05 of statistically significant contrasts as revealed by *post-hoc* tests **(B)** AUC scores across participants in the KNN model show a low amount of inter-individual variance. In this figure, the blocking variable “Participant” is accounted for. Methods indicated as follows: MA, motor area ROI; ICA, independent component analysis; WB, whole-brain ROI; MAN, manual artifact correction.

Applying the SVM model, there was no significant influence of the artifact removal [*F*_(1,9.36)_ = 0.024, *p* = 0.881] or of ROI [*F*_(1,9.858)_ = 0.064, *p* = 0.806] on AUC, (see Figures 6A, B). In the KNN model with *k* = 10, the effect of artifact removal on AUC values was not significant [*F*_(1,9.998)_ = 0.806, *p* = 0.390]. However, the effect of ROI on AUC scores was statistically significant [*F*_(1,8.939)_ = 7.585, *p* = 0.023, see Figures 7A, B]. The Conover *post-hoc* (Conover, 1998) (R package “PMCMR,” Pohlert, 2018), which is based on the *Friedman* ANOVA as an alternative to the robust ANOVA, was used to identify the combinations of predictor levels, which contributed to the significant effect in the *robust* ANOVA. The test revealed significant differences among three specific combinations of ROI and artifact cleaning: “Motor Area and ICA artifact correction” vs. “Whole Brain Area and ICA artifact correction” (*p* = 0.015), “Motor Area and ICA artifact correction” vs. “Whole Brain Area and manual correction” (*p* = 0.021), and “Motor Area and manual correction” vs. “Whole Brain Area and ICA correction” (*p* = 0.027). All reported *p*-values were FDR-corrected. The combination of “Motor Area and ICA artifact correction” returned the highest AUC, followed by “Motor Area and manual correction,” “Whole Brain Area and ICA artifact correction,” and “Whole Brain Area and manual artifact correction.”

For the SVM classifier model, there was no significant effect of the artifact cleaning on brier scores [*F*_(1,9.690)_ = 0.531; *p* = 0.484], and also no significant effect of the electrodes space [*F*_(1,9.262)_ = 3.705; *p* = 0.086] on brier scores. For the KNN classifier model, there was no significant effect of the artifact cleaning on brier scores [*F*_(1,7.924)_ = 0.084; *p* = 0.779], and also no significant effect of the electrodes space [*F*_(1,7.188)_ = 0.958; *p* = 0.360].

AUC and brier score values over the four different preprocessing methods applied per participant can be found in Table 1 for the SVM model and in Table 2 for the KNN model.

### 3.3. Correctly classified instances per preprocessing condition (RQ3)

Determining correctly classified instances were based on *p*-values from the single-subject binomial tests (mental MI vs. resting-state) as indicated in Section 2. For participant “S8,” classification could not be done due to excessive artifacts in the manual artifact correction, resulting in only two models for this participant. For the SVM model, the method “motor area with ICA” produced 6 out of 8 models (75%) with significant classifications, “motor area with manual artifact correction” 7 out of 7 models (100%), “whole brain area with ICA” 6 out of 8 models (75%) and “whole brain area with manual artifact correction” 5 out of 7 (71%) success rate (as indicated in Table 1). In the KNN model, with the preprocessing method “motor area with ICA” 3 out of 8 models (38%) resulted in a significant classification of mental MI vs. resting-state tasks, with “motor area with manual artifact correction” 2 out of 7 models (29%), with “whole brain area with ICA” 1 out of 8 models (13%), and “whole brain area with manual artifact correction” 0 out of 7 models (0%). Thus, the probability for a correct detection of mental MI and resting-state patterns across participants ranged from 63 to 88% for the SVM algorithm and from 0 to 38% for the KNN algorithm (as listed in Table 2).

### 3.4. Trends in order of starting block (RQ4a and 4b)

**Figure 8:**
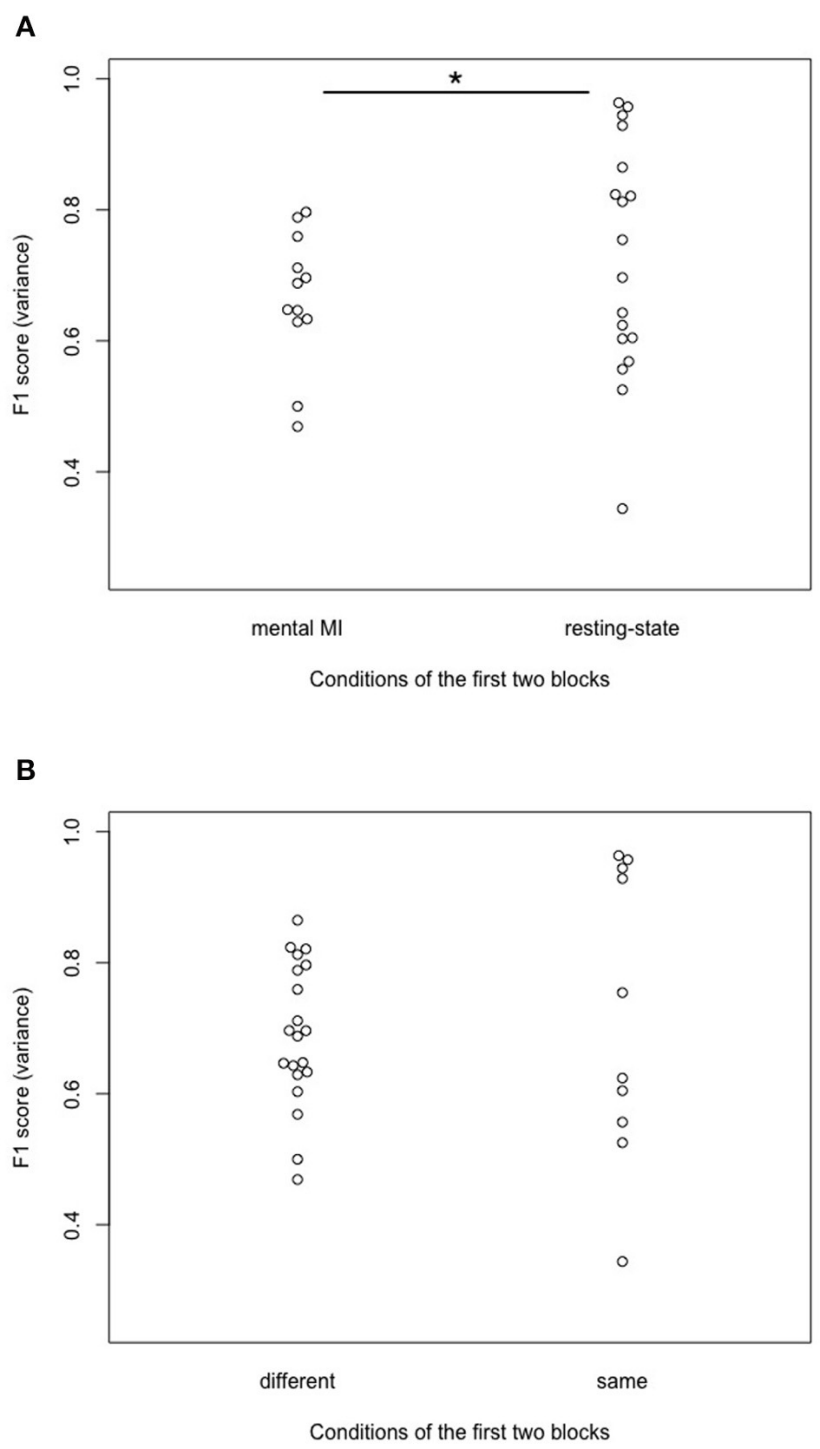
Effects of the configuration of the first two blocks on predicting participant performance. **(A)** Significant effect of the condition of the first block of the experiment on variance in predicting participant performance (F1 variance) after the first two mental MI and resting-state blocks. Start condition “mental MI” and “resting-state” indicate with which condition the randomized block order started for a participant. * Denotes statistically significant difference at 95% level **(B)** No effect of the configuration of the first two experimental blocks on variance (F1 variance) in predicting participant performance after the first two mental MI and resting-state blocks. Start condition “different” indicates cases where participants were presented one mental MI and one resting-state block at the beginning; “same” indicates cases where participants were presented either two mental MI or resting-state blocks, respectively.

In an exploratory way, we looked at the difference in model quality (F1) variance, depending on the task at the beginning of the experiment (see Supplementary Table 2). Variance was chosen instead of the mean since it respects the individual performance more than the mean. It was investigated, for example, whether starting with a resting-state block would facilitate the mental imagination in participants, compared with starting with a mental MI block. Thus, we looked at differences in variance between participants starting with mental MI vs. resting-state in the first block presented, using a Fligner-Killeen test on variance differences. Participants starting the complete procedure with a resting-state block showed significantly higher F1 scores variances compared to starting with a mental MI block [*X*^2^_(1)_ = 5.849, *p* = 0.016, see Figure 8A]. Expanding this approach, it was also investigated if starting the experiment with two different conditions in the first two blocks (e.g., mental MI—resting-state; resting-state—mental MI) differed from the same condition delivered in these blocks (e.g., resting-state—resting-state; mental MI—mental MI) in terms of F1 score variance. There was no significant difference between these configurations in F1 score variance [*X*^2^_(1)_ = 1.339, *p* = 0.247, see Figure 8B].

## 4. Discussion

There was no significant effect of signal-processing method on F1, indicating participant performance, irrespective of the machine-learning model used (SVM model with a linear kernel and fold optimization in the cross-validation or KNN model with optimization of neighbors). In the words of RQ1: the representation of participant performance was not influenced significantly by the signal-processing applied to the EEG data. AUC scores from ROC analyses were not influenced by signal preprocessing in the SVM model, but they were influenced by preprocessing in the KNN model. In the framework of RQ2: Signal-processing affected the quality of the machine-learning algorithms only in the KNN models, not in the SVM classifier models. Artifact cleaning and electrodes space selection did not influence model calibration scores (brier scores), neither in the SVM nor in the KNN classifier model. Furthermore, in RQ4a we looked at the variance in participant performance related to the first block of tasks (mental MI vs. resting-state). The variance in the performance scores was significantly higher in participants who started the experiment with a resting-state task block (F1 scores reached from under 0.4 to almost 1.0) compared to a mental MI task block (F1 cores ranging from 0.4 to 0.8).

To the best of our knowledge, a comparable approach to investigate EEG signal preprocessing strategies and different classifiers in a mental MI classification setting has not been done before, and literature is lacking a clear consensus on how to deal with EEG signal preprocessing, ROI selection and classifier selection. Henriques et al. (2016) varied the field of electrodes together with the signal extraction method (Fourier method vs. parametric model), finding that a larger electrodes set performed better than a smaller set. Höller et al. (2013a) found the same effect of electrodes set size. Actually, the current trend in literature is a down-sizing of the number of electrodes to standard 21-electrodes montage (Claassen et al., 2019) or three electrodes (Coyle et al., 2015). However, focusing on a minimal number of electrodes over a central region might miss important activity of other brain areas involved in mental MI. Imagining oneself swimming and concentrating on the motion itself is intended to activate the motor cortex. Still, the prefrontal cortex (planning) or the visual cortex might be activated as well by the instruction. Analyzing the whole brain's activity thus seems straight-forward. As Höller et al. (2013a) and Henriques et al. (2016) found a more correctly classified instances of mental MI in larger electrodes sets, it can be assumed that apart from the central region over the primary motor cortex further brain regions might be relevant. This could not be shown in the trial-based analysis of this study, neither in the SVM, nor in the KNN model. The reason why this effect was missed in this work could be due to the fact that the choice of artifact rejection was directly linked to the choice of the electrodes set and thus was hiding the effect.

In the end, the more sophisticated preprocessing approach combining manual artifact rejection before and after an ICA, as applied in more recent literature (Chennu et al., 2017) did not result in a better representation of participant performance than just removing artifacted trials and EEG channels from the analysis as applied in some publications on MI (Cruse et al., 2011, 2012a,b). The ICA approach had been chosen to remove any particular ocular, myogenic, or myographic artifacts, which could not be removed by solely removing contaminated channels and trials. Due to a back-transformation into time-frequency space (Höller et al., 2013a), no additional channels or trials were removed by the ICA itself. As reported in Section 2.4, the number of trials removed in the smaller ROI over M1 was not significantly different from the number of trials removed in the whole-brain ROI. Generally, data sets had not the same number of good trials included (see Supplementary Table 1). Models with ICA-based artifact removal had significantly less good trials than models with manual artifact removal. It seems that with the second manual removal procedure after ICA, data was further artificially reduced. Thus, the number of included trials in the process of artifact removal could be an important confounding factor for the classification of mental MI and resting-state tasks, leading to the observed variability in the outcomes (representation of participant performance and classifier performance). Artifact correction only with an ICA (no manual removal of trials or channels) was not tested in this study, as it had shown no gain in clean signal over manual artifact correction in a study by Horki et al. (2014). For patients' data, the authors recommend an artifact identification and removal procedure combining thresholds for bad channels/trials and an ICA, which was adopted in this protocol, in the prospect of future work on patients' data sets.

Single-subject analysis revealed that for many of the classification models, the quality of the separation between mental MI and resting-state tasks was not significantly better than chance. On a group level, cases where the classifier was operating significantly better than chance level in correctly detecting instances of mental MI and resting-state tasks was higher for the SVM-based models (between 71 and 100%) than for the KNN models (between 0 and 38%). So, probably the SVM algorithm was better able to correctly classify brain patterns reflecting mental MI and resting-state than the KNN algorithm. Notably, the accuracy scores (F1) representing participant performance values were not perfect in any models: In all SVM models where the classifier was operating significantly better than chance level in correctly detecting instances of mental MI and resting-state tasks, the accuracy values ranged from 60.8 to 96.4%, in all KNN models between 62.0 and 67.0% (see Tables 1, 2). These results are consistent with the literature: in a comparable study 75% of all healthy controls were able to produce MI brain patterns, which could be distinguished statistically from each other, and of these healthy controls who managed to follow commands (9 out of 12), the classification accuracies ranged between 60 and 91% (Cruse et al., 2011). In a follow-up project (Cruse et al., 2012b), the authors changed the task to only include imagined movements of the left or right hand and reduced the EEG registration to four central electrodes. The paradigm was tested in a chronic UWS patient successfully; however, only 5 out of 6 (83%) of healthy controls were able to produce EEG patterns significantly distinguishing left-hand from right-hand imagined movements on command with a median classification accuracy of 67% (range 60–80%). These accuracies were reached using a naive Bayes classifier, SVM-based accuracy values obtained from our healthy controls sample are even higher. Yet, this work fail to be perfect in the sense of detecting patterns that map participant behavior to 100% accuracy. This certainly presents a severe threat to validity. As outlined above, prior work can only give an estimate of good performance values, and stable performance values are hard to obtain. Bad classification could be linked to the quality of the participant's covert mental responses to the tasks (i.e., mental MI and resting-state), which are participant-dependent and thus a-priori unknown. The classifier tries to obtain the most important (e.g., recurrent) features from highly individual EEG signal, which underlies natural variability and is never fully predictable.

The high inter-subject variability in F1 and AUC scores found in this study resembles the results obtained by Henriques et al. (2016) and Goldfine et al. (2011). Henriques et al. (2016) mentioned that the order of task execution could influence the signal detection. Exploratory, we looked at mechanisms which could have produced this inter-subject variance, e.g., sequence of tasks and task difficulty. On a trial-level, we investigated whether or not starting with a block resting-state tasks followed by a block of mental MI tasks or vice versa had an effect on participants' performance (variance in F1 scores). There was a significant difference in F1 score variance if participants started with a resting-state task block instead of a mental MI task block (see Figure 8A). Thus, the variance in the classifier detecting mental command-following was lower when participants started with a MI sequence, compared to a resting-state sequence. Starting with a resting-state sequence seemed to display the participants' ability for command-following more detailed than starting the experiment with a MI sequence. Such effects of block order in the presentation of the tasks were also taken into account by Cruse et al. (2011) and Cruse et al. (2012a,b), who chose a pseudo-randomization of task blocks. According to the authors, pseudo-randomization should ensure that dependencies within and between blocks were reduced to a minimum and thus did not affect performance (Cruse et al., 2011). To suppress such inter- and intrablock dependence in this study, blocks were fully randomized. Going further, we additionally investigated whether two blocks of the same task caused a significantly different outcome than two blocks of different tasks at the beginning of the experiment. Starting with two resting-state or mental MI task blocks was not associated with significantly higher inter-subject variance in F1 scores compared to starting with blocks from different tasks (see Figure 8B). Regarding intrablock-dependence (Henriques et al., 2016) this means that two adjacent blocks of the same condition at the beginning of the experiment did not facilitate classification compared to two different conditions (see Figure 8B), and also did not affect trial-based prediction of participant performance. Thus, adjacency of blocks at the beginning of the experiment was unlikely to have contributed to the inter-subject variability in classification in the present work. As the block-structure was broken in the trial-based machine-learning analyses, the order of block presentation might not be the only valid explanation for inter-subject variability. The number of trials might be another explanation for variability of participant performance scores on the single-subject level. This explanation could be ruled out, as the number of included trials per model and the participant performance scores did not correlate significantly with each other, neither in the models with SVM classification, nor with KNN classification.

Besides the sequence of tasks, we expected the task's cognitive load to account for variability in classification. As Henriques et al. (2016) mentioned, only a diagnostic method with high sensitivity and specificity in healthy controls is ready for use in patients with DOC. Thus, the task should be easy to solve for detecting as many true positives as possible. Obviously, the less participants have to switch between blocks of different conditions the easier a task becomes. Switching between trials of different tasks is harder then sticking to one task for an entire block of trials (Horki et al., 2014). In the present study there was no significant difference in the variance of predicting participant performance scores (F1) if the tasks in the first tow blocks were the same or not, so switching tasks after only one block was not harder for the classification process than the same task for two consecutive blocks. Additionally, it has to be taken into account that all participants reported to be unfamiliar with mental MI. After the experiment, some participants mentioned that they had difficulties executing the mental MI task without exercise or adjustment to the surroundings. Inspired by these comments, we looked at participants who had started with a mental MI block vs. resting-state block. Indeed, we found a significantly higher variance in mental MI performance scores (F1) for participants starting with a resting-state block, no matter of the experimental condition of the following task blocks (Figure 8A). Starting the experiment with a resting-state block could help participants to become familiar with the experiment and the lab surroundings. However, it has to be taken into account that data from eight participants is not enough to draw proper conclusions. For an ideal classifier performance, tasks should be on the one hand complex, and on the other hand familiar (Gibson et al., 2014). Familiarity is also mentioned by Henriques et al. (2016) as an important prerequisite for successful classification, together with salient stimuli. A particular mental MI task is probably not suited to all participants (Mohamed et al., 2018). The mental MI task in this study tried to fulfill these recommendations for a good MI task, as it was on the one hand more engaging and complex than, e.g., hand movements, and also a culturally transmitted task that should be familiar to many people.

The protocol also comes along with limitations: The present work only compared a limited amount of signal processing methods and classifiers that are currently available. Therefore, we can only give a limited answer to the research questions regarding the overall effect of signal processing. The sample size of the study was small, limiting the power to detect a statistically meaningful effect. Reduced power could lead to false-negative findings (e.g., in this case poor KNN classifier performance). Further testing in bigger sample sizes would be necessary to address this limitation and to answer the second research question on sequence effects of block sequence more in-depth. Future studies could test other existing classifiers to address the generalizability of the results [e.g., linear discriminant analysis, common spatial patterns (Fu et al., 2020), or successive decomposition indices (Sadiq et al., 2020)]. The complete randomization of blocks was chosen to avoid intra-block dependency, however it might have caused the high variability in performance values across participants. A design with a fixed order of task blocks could exclude potential influences of the task order that lead to an overly optimistic prediction of participant or classifier performance. Further, in one participant, excessive artifacts in the signal made the application of a purely manual artifact correction (without ICA) impossible. This case justifies the additional value of an ICA in case of artifact-ridden data, as it removes artifacts more carefully than a deletion of entire sections of data. On a group level, with the ICA procedure significantly more trials had been removed compared to manual artifact removal. This might have been caused by the additional manual correction after the ICA. In future work, one iteration of manual artifact removal plus ICA might be a good way not to delete too much data and still keep the in-depth removal of noisy signal. It is of course impossible to infer from EEG signal that participants actually imagined themselves swimming or if they just listened to the auditory triggers. The SVM and KNN model trained on the EEG features was supposed to discriminate between two different brain states, but it cannot serve as a “mind-reader.” Thus, it is beyond the scope of this work to draw any conclusions on the actual content of thoughts. The intention of a mental MI task proving for command-following is to test if participants can repeatedly and consciously manipulate their brain activity successfully. Of course, participants could engage in more than two brain states during the EEG session, which is not modeled by the algorithm. Consequently, the SVM would try to classify these deviating brain states to the known imagery classes. Hidden Markov Models could be an alternative for future studies interested in capturing of multiple brain states. Additionally, EEG signals present a low signal-to-noise ratio, making them prone to the phenomenon of volume conduction. Thus, conclusions regarding the underlying neural mechanisms are difficult to draw (Lu et al., 2016). Source localization in scalp EEGs is a possible way to localize changes in EEG patterns (Qin et al., 2004).

The present study sought to determine the role of signal preprocessing (e.g., choice of electrode space and method for artifact rejection) and machine-learning approaches on the prediction of participant performance and classifier performance in an EEG-based mental MI paradigm in healthy adults. Effects of signal preprocessing procedures on predicting participant performance (F1 scores) could not be found, neither in the SVM nor the KNN model. Importantly, the KNN model classifier performance (AUC scores) was influenced by the signal preprocessing (electrode space selection), compared the SVM model. Exploratory analyses gave a hint toward potential effects of task sequence in the protocol design on the prediction of participant performance: a resting-state task block at the beginning of the experiment seems to ease mental command-following detection. The analyses helped to gain insight into important mechanisms to consider, such as the choice of electrodes space, artifact removal technique, classifier type, and task sequence when designing and analyzing EEG-based mental MI paradigms. Choices should be made thoroughly and be based on careful considerations of the experiment design.

## Data availability statement

The raw data supporting the conclusions of this article will be made available by the authors, without undue reservation.

## Ethics statement

The studies involving human participants were reviewed and approved by Ethics Committee of the Medical Faculty of the University of Munich. The patients/participants provided their written informed consent to participate in this study.

## Author contributions

MR and MS designed the experiment(s). MR conducted the experiment. MR and MP analyzed the EEG data. BH and MR conducted the machine-learning analysis of the results. MR wrote the manuscript. MR, RP, MS, and PF contributed critically to the design of the study. All authors reviewed the manuscript.

## References

[B1] Aggarwal S. Chugh N. (2019). Signal processing techniques for motor imagery brain computer interface: a review. Array 1:100003. 10.1016/j.array.2019.100003

[B2] Aggarwal S. Chugh N. (2022). Review of machine learning techniques for EEG based brain computer interface. Arch. Computat. Methods Eng. 29, 3001–3020. 10.1007/s11831-021-09684-6

[B3] Ballings M. Van den Poel D. (2013). AUC: a grammar of data manipulation. Package AUC. Available online at: http://cran.r-project.org/web/packages/AUC/AUC.pdf (accessed April 15, 2023).

[B4] Bell A. J. Sejnowski T. J. (1995). An information-maximization approach to blind separation and blind deconvolution. Neural Comput. 7, 1129–1159. 10.1162/neco.1995.7.6.1129 7584893

[B5] Chennu S. Annen J. Wannez S. Thibaut A. Chatelle C. Cassol H.. (2017). Brain networks predict metabolism, diagnosis and prognosis at the bedside in disorders of consciousness. Brain 140, 2120–2132. 10.1093/brain/awx163 28666351

[B6] Claassen J. Doyle K. Matory A. Couch C. Burger K. M. Velazquez A.. (2019). Detection of brain activation in unresponsive patients with acute brain injury. N. Engl. J. Med. 380, 2497–2505. 10.1056/NEJMoa1812757 31242361

[B7] Conover W. J. (1998). Practical Nonparametric Statistics, Vol. 350. New York, NY: John Wiley & Sons.

[B8] Coyle D. Stow J. McCreadie K. McElligott J. Carroll Á. (2015). Sensorimotor modulation assessment and brain-computer interface training in disorders of consciousness. Arch. Phys. Med. Rehabil. 96, S62–S70. 10.1016/j.apmr.2014.08.024 25721549

[B9] Craik A. He Y. Contreras-Vidal J. L. (2019). Deep learning for electroencephalogram (eeg) classification tasks: a review. J. Neural Eng. 16:031001. 10.1088/1741-2552/ab0ab5 30808014

[B10] Cruse D. Chennu S. Chatelle C. Bekinschtein T. A. Fernández-Espejo D. Pickard J. D.. (2011). Bedside detection of awareness in the vegetative state: a cohort study. Lancet 378, 2088–2094. 10.1016/S0140-6736(11)61224-5 22078855

[B11] Cruse D. Chennu S. Chatelle C. Fernández-Espejo D. Bekinschtein T. A. Pickard J. D.. (2012a). Relationship between etiology and covert cognition in the minimally conscious state. Neurology 78, 816–822. 10.1212/WNL.0b013e318249f764 22377810 PMC3304945

[B12] Cruse D. Chennu S. Fernández-Espejo D. Payne W. L. Young G. B. Owen A. M. (2012b). Detecting awareness in the vegetative state: electroencephalographic evidence for attempted movements to command. PLoS ONE 7:e49933. 10.1371/journal.pone.0049933 23185489 PMC3503880

[B13] Engemann D. A. Raimondo F. King J.-R. Rohaut B. Louppe G. Faugeras F.. (2018). Robust EEG-based cross-site and cross-protocol classification of states of consciousness. Brain 141, 3179–3192. 10.1093/brain/awy251 30285102

[B14] Fu R. Han M. Tian Y. Shi P. (2020). Improvement motor imagery EEG classification based on sparse common spatial pattern and regularized discriminant analysis. J. Neurosci. Methods 343:108833. 10.1016/j.jneumeth.2020.108833 32619588

[B15] Gibson R. M. Chennu S. Owen A. M. Cruse D. (2014). Complexity and familiarity enhance single-trial detectability of imagined movements with electroencephalography. Clin. Neurophysiol. 125, 1556–1567. 10.1016/j.clinph.2013.11.034 24388403

[B16] Goldfine A. M. Bardin J. C. Noirhomme Q. Fins J. J. Schiff N. D. Victor J. D. (2013). Reanalysis of “bedside detection of awareness in the vegetative state: a cohort study”. Lancet 381, 289–291. 10.1016/S0140-6736(13)60125-7 23351802 PMC3641526

[B17] Goldfine A. M. Victor J. D. Conte M. M. Bardin J. C. Schiff N. D. (2011). Determination of awareness in patients with severe brain injury using EEG power spectral analysis. Clin. Neurophysiol. 122, 2157–2168. 10.1016/j.clinph.2011.03.022 21514214 PMC3162107

[B18] Henriques J. Gabriel D. Grigoryeva L. Haffen E. Moulin T. Aubry R.. (2016). Protocol design challenges in the detection of awareness in aware subjects using EEG signals. Clin. EEG Neurosci. 47, 266–275. 10.1177/1550059414560397 25488924

[B19] Höller Y. Bergmann J. Kronbichler M. Crone J. S. Schmid E. V. Thomschewski A.. (2013a). Real movement vs. motor imagery in healthy subjects. Int. J. Psychophysiol. 87, 35–41. 10.1016/j.ijpsycho.2012.10.015 23123181

[B20] Höller Y. Bergmann J. Thomschewski A. Kronbichler M. Höller P. Crone J. S.. (2013b). Comparison of eeg-features and classification methods for motor imagery in patients with disorders of consciousness. PLoS ONE 8:e80479. 10.1371/journal.pone.0080479 24282545 PMC3839976

[B21] Horki P. Bauernfeind G. Klobassa D. S. Pokorny C. Pichler G. Schippinger W.. (2014). Detection of mental imagery and attempted movements in patients with disorders of consciousness using EEG. Front. Hum. Neurosci. 8:1009. 10.3389/fnhum.2014.01009 25566029 PMC4264500

[B22] Jung T.-P. Makeig S. Humphries C. Lee T.-W. Mckeown M. J. Iragui V.. (2000). Removing electroencephalographic artifacts by blind source separation. Psychophysiology 37, 163–178. 10.1111/1469-8986.3720163 10731767

[B23] Krusienski D. J. McFarland D. J. Wolpaw J. R. (2012). Value of amplitude, phase, and coherence features for a sensorimotor rhythm-based brain-computer interface. Brain Res. Bull. 87, 130–134. 10.1016/j.brainresbull.2011.09.019 21985984 PMC3246076

[B24] Liu J. Ye F. Xiong H. (2021). Multi-class motor imagery EEG classification method with high accuracy and low individual differences based on hybrid neural network. J. Neural Eng. 18:0460f1. 10.1088/1741-2552/ac1ed0 34407527

[B25] Lomelin-Ibarra V. A. Gutierrez-Rodriguez A. E. Cantoral-Ceballos J. A. (2022). Motor imagery analysis from extensive EEG data representations using convolutional neural networks. Sensors 22:6093. 10.3390/s22166093 36015854 PMC9414220

[B26] Lu N. Li T. Ren X. Miao H. (2016). A deep learning scheme for motor imagery classification based on restricted Boltzmann machines. IEEE Trans. Neural Syst. Rehabil. Eng. 25, 566–576. 10.1109/TNSRE.2016.2601240 27542114

[B27] Mahmoudi M. Shamsi M. (2018). Multi-class EEG classification of motor imagery signal by finding optimal time segments and features using SNR-based mutual information. Austr. Phys. Eng. Sci. Med. 41, 957–972. 10.1007/s13246-018-0691-2 30338495

[B28] Makeig S. Jung T.-P. Ghahremani D. Sejnowski T. J. (1996). Independent Component Analysis of Simulated ERP Data. Technical Report INC-9606, Institute for Neural Computation, University of California.

[B29] MATLAB (2017). MATLAB (R2017a), Version 9.2.0. Natick, MA: The MathWorks Inc.

[B30] McMenamin B. W. Shackman A. J. Maxwell J. S. Bachhuber D. R. Koppenhaver A. M. Greischar L. L.. (2010). Validation of ICA-based myogenic artifact correction for scalp and source-localized EEG. Neuroimage 49, 2416–2432. 10.1016/j.neuroimage.2009.10.010 19833218 PMC2818255

[B31] Milton J. Small S. L. Solodkin A. (2008). Imaging motor imagery: methodological issues related to expertise. Methods 45, 336–341. 10.1016/j.ymeth.2008.05.002 18762138 PMC5536170

[B32] Mohamed E. A. Yusoff M. Z. Malik A. S. Bahloul M. R. Adam D. M. Adam I. K. (2018). Comparison of EEG signal decomposition methods in classification of motor-imagery BCI. Multimedia Tools Appl. 77, 21305–21327. 10.1007/s11042-017-5586-9

[B33] Nguyen C. N. Zeigermann O. (2018). Machine Learning-Kurz & *Gut: Eine Einführung mit Python*. O'Reilly: Pandas und Scikit-Learn.

[B34] Olbrich S. Jödicke J. Sander C. Himmerich H. Hegerl U. (2011). ICA-based muscle artefact correction of EEG data: what is muscle and what is brain?: Comment on Mcmenamin et al. Neuroimage 54, 1–3. 10.1016/j.neuroimage.2010.04.256 20441799

[B35] Oostenveld R. Fries P. Maris E. Schoffelen J.-M. (2011). Fieldtrip: open source software for advanced analysis of MEG, EEG, and invasive electrophysiological data. Comput. Intell. Neurosci. 2011:156869. 10.1155/2011/156869 21253357 PMC3021840

[B36] Pfurtscheller G. Scherer R. Müller-Putz G. Lopes da Silva F. (2008). Short-lived brain state after cued motor imagery in naive subjects. Eur. J. Neurosci. 28, 1419–1426. 10.1111/j.1460-9568.2008.06441.x 18973568

[B37] Pohlert T. (2018). Calculate Pairwise Multiple Comparisons of Mean Rank Sums. Package ‘pmcmr'. R package version, 1(0). Available online at: https://cran.microsoft.com/snapshot/2020-04-20/web/packages/PMCMR/PMCMR.pdf (accessed April 15, 2023).

[B38] Qin L. Ding L. He B. (2004). Motor imagery classification by means of source analysis for brain-computer interface applications. J. Neural Eng. 1:135. 10.1088/1741-2560/1/3/002 15876632 PMC1945182

[B39] R Core Team (2019). The R Stats Package. R Foundation for Statistical Computing, Vienna, Austria. Available online at: https://www.R-project.org

[B40] RStudio Team (2020). RStudio: Integrated Development Environment for R. Boston, MA: RStudio, PBC.

[B41] Sadiq M. T. Yu X. Yuan Z. Aziz M. Z. (2020). Identification of motor and mental imagery EEG in two and multiclass subject-dependent tasks using successive decomposition index. Sensors 20:5283. 10.3390/s20185283 32947766 PMC7570740

[B42] Schack T. Essig K. Frank C. Koester D. (2014). Mental representation and motor imagery training. Front. Hum. Neurosci. 8:328. 10.3389/fnhum.2014.00328 24904368 PMC4033090

[B43] Stender J. Gosseries O. Bruno M.-A. Charland-Verville V. Vanhaudenhuyse A. Demertzi A.. (2014). Diagnostic precision of pet imaging and functional MRI in disorders of consciousness: a clinical validation study. Lancet 384, 514–522. 10.1016/S0140-6736(14)60042-8 24746174

[B44] Zhang H. Zhao X. Wu Z. Sun B. Li T. (2021). Motor imagery recognition with automatic EEG channel selection and deep learning. J. Neural Eng. 18:016004. 10.1088/1741-2552/abca16 33181505

