# Supplementary material for: Stability of mental motor-imagery classification in EEG depends on the choice of classifier model and experiment design, but not on signal preprocessing
Source: Front Comput Neurosci. 2023 Apr 26;17:1142948. doi: 10.3389/fncom.2023.1142948 (PMC10169631; doi:10.3389/fncom.2023.1142948)
Supplement: Supplementary file 1 [file Data_Sheet_1.pdf]

Supplementary Material

1 SUPPLEMENTARY DATA

$$\text{Accuracy} = \frac{TruePositives + TrueNegatives}{TruePositives + TrueNegatives + FalsePositives + FalseNegatives} \tag{S1}$$

$$\text{F1} = 2 \cdot \frac{2 \cdot TruePositives}{2 \cdot TruePositives + FalsePositives + FalseNegatives} \tag{S2}$$

$$\text{Precision} = \frac{TruePositives}{TruePositives + FalsePositives} \tag{S3}$$

$$\text{Sensitivity} = \frac{TruePositives}{TruePositives + FalseNegatives} \tag{S4}$$

$$\text{Specificity} = \frac{TrueNegatives}{TrueNegatives + FalsePositives} \tag{S5}$$

Supplementary Data S1-S5 Formulae used to calculate participant performance scores (accuracy and F1) and model characteristics (sensitivity and specificity).

2 SUPPLEMENTARY TABLES AND FIGURES

2.1 Tables

Table S1. Overview of EEG features per dataset. ICA = Independent Component Analysis, MA = Motor Area, MAN = Manual Artifact Detection, ROI = Region of Interest, WB = Whole-Brain Area, Channels = number of channels in the dataset, trials = number of trials in the complete dataset, 'Mental MI' trials = number of trials in the Motor Imagery condition, 'resting-state' trials = number of trials in the resting-state condition.

Participant	ROI	Cleaning	Channels	Trials	'Mental MI' trials	'resting-state' trials
P1	MA	ICA	33	118	59	59
P2	MA	ICA	29	94	52	42
P3	MA	ICA	23	113	59	54
P4	MA	ICA	27	104	47	57
P5	MA	ICA	33	118	59	59
P6	MA	ICA	29	111	53	58
P7	MA	ICA	23	108	50	58
P8	MA	ICA	28	108	57	51
P1	MA	MAN	33	118	59	59
P2	MA	MAN	31	108	55	53
P3	MA	MAN	31	115	59	56
P4	MA	MAN	28	114	54	60
P5	MA	MAN	33	120	60	60
P6	MA	MAN	30	115	57	58
P7	MA	MAN	30	105	49	56
P8	MA	MAN	NA	NA	NA	NA
P1	WB	ICA	157	109	56	53
P2	WB	ICA	156	95	53	42
P3	WB	ICA	155	114	55	59
P4	WB	ICA	160	105	51	54
P5	WB	ICA	165	105	53	52
P6	WB	ICA	160	105	52	53
P7	WB	ICA	168	98	46	52
P8	WB	ICA	171	101	60	41
P1	WB	MAN	158	112	56	56
P2	WB	MAN	159	106	54	52
P3	WB	MAN	152	110	54	56
P4	WB	MAN	152	110	55	55
P5	WB	MAN	168	112	57	55
P6	WB	MAN	159	114	56	58
P7	WB	MAN	168	107	49	58
P8	WB	MAN	NA	NA	NA	NA

Table S2. Representation of block order configurations for all participants. Every participant was presented six blocks in randomized order of which three blocks were 'mental MI' and 'resting-state', respectively.

Participant	Block 1	Block 2	Block 3	Block 4	Block 5	Block 6
P1	Mental MI	Mental MI	Mental MI	resting-state	resting-state	resting-state
P2	Mental MI	Mental MI	Mental MI	resting-state	resting-state	resting-state
P3	resting-state	Mental MI	Mental MI	Mental MI	resting-state	resting-state
P4	resting-state	resting-state	Mental MI	Mental MI	Mental MI	resting-state
P5	Mental MI	resting-state	Mental MI	Mental MI	resting-state	resting-state
P6	resting-state	resting-state	Mental MI	Mental MI	resting-state	Mental MI
P7	resting-state	Mental MI	Mental MI	resting-state	Mental MI	resting-state
P8	resting-state	resting-state	Mental MI	Mental MI	resting-state	Mental MI

Table S3. Combinations of experimental settings for the analyses with all six blocks. Manual = manual artifact cleaning, ICA = Independent Component Analysis, SVM = support vector machine, KNN = k-nearest-neighbors.

Electrode set	motor area	whole-brain
Artifact correction	manual / ICA + manual	manual / ICA + manual
Folds crossvalidation	10	10
Crossvalidation model	SVM / KNN	SVM / KNN

Table S4. Variation of hyperparameters in the support vector machine (SVM) model for single-subject classification. F1 scores (participant performance) are given for all hyperparameter constellations as mean and standard deviation (SD) across all participants. For every region of interest (ROI) and artifact cleaning method the best mean F1 score is written in bold letters. As 10 folds had the best mean F1 score in three out of four constellations, 10 folds were chosen for all classifiers in the single-subject classification. MAN = manual artifact cleaning, ICA = Independent Component Analysis.

ROI	Cleaning	Cross-validation folds	Mean	SD
Motor Area	ICA	5	0,653144965	0,150227239
		10	**0,70425**	0,132615751
		20	0,654262723	0,147902019
	MAN	5	0,692541168	0,168702279
		10	**0,726342857**	0,132484391
		20	0,693620598	0,169325348
Whole Brain	ICA	5	0,686124241	0,162987705
		10	**0,7041**	0,144225899
		20	0,68467977	0,150478393
	MAN	5	**0,692944757**	0,140769781
		10	0,664371429	0,180672654
		20	0,685844445	0,148044121

Table S5. Variation of hyperparameters in the k-nearest-neighbors (KNN) model for single-subject classification. F1 scores (participant performance) are given for all hyperparameter constellations as mean and standard deviation (SD) across all participants. For every region of interest (ROI) and artifact cleaning method the best mean F1 score is written in bold letters. As 10 folds had the best mean F1 score in most of the constellations (8/20), 10 folds were chosen for all classifiers in the single-subject classification. MAN = manual artifact cleaning, ICA = Independent Component Analysis.

ROI	Cleaning	neighbors	Cross-validation folds	Mean	SD
Motor Area	ICA	1	5	0,575661422	0,135838618
			10	0,575681382	0,128865897
			20	**0,575690575**	0,129826028
		3	5	0,562989225	0,129166804
			10	**0,572544997**	0,128265611
			20	0,567537988	0,130933239
		5	5	**0,575598887**	0,141290825
			10	0,56268981	0,14439144
			20	0,570812667	0,140463773
		10	5	0,595514043	0,128916054
			10	**0,6068125**	0,145919125
			20	0,588973501	0,119367838
		15	5	**0,545127932**	0,170497213
			10	0,539024101	0,160827297
			20	0,540159148	0,152641091
Motor Area	MAN	1	5	**0,602496623**	0,09960511
			10	0,600567052	0,108615378
			20	0,599503768	0,103370763
		3	5	0,580473935	0,114018392
			10	0,576699941	0,115092606
			20	**0,587482865**	0,115238457
		5	5	0,556072924	0,128570403
			10	**0,557279733**	0,136412042
			20	0,555336644	0,136890446
		10	5	0,551317559	0,135540492
			10	**0,594728571**	0,107777293
			20	0,560101257	0,122363598
		15	5	0,507884135	0,17523686
			10	**0,514387731**	0,177110627
			20	0,510527947	0,181701239
Whole Brain	ICA	1	5	**0,606572065**	0,107967896
			10	0,602156743	0,103850337
			20	0,596859843	0,104433954
		3	5	0,580535769	0,134377486
			10	0,580890481	0,134326281
			20	**0,582444919**	0,131191129
		5	5	0,584551852	0,146577952
			10	0,582939659	0,137095183
			20	**0,585487041**	0,141579235
		10	5	0,582783017	0,125719356
			10	**0,6007**	0,135895456
			20	0,594997898	0,127251571
		15	5	**0,561417345**	0,167667908
			10	0,558915662	0,189030948
			20	0,555022256	0,181582802
Whole Brain	MAN	1	5	0,612121551	0,106088799
			10	0,611229392	0,103053558
			20	**0,61277809**	0,107838232
		3	5	0,60039421	0,079942713
			10	0,602460507	0,088581883
			20	**0,609024033**	0,088796169
		5	5	0,596724241	0,095343586
			10	0,602878207	0,090790076
			20	**0,611147095**	0,100768171
		10	5	0,597677477	0,09572731
			10	**0,602771429**	0,094412162
			20	0,597860553	0,1028744
		15	5	0,564865707	0,127951052
			10	**0,571781849**	0,124851302
			20	0,569888082	0,125693992
